# Integrated NMR, HPLC-Chemometrics, and Network Pharmacology Strategy for Quality Assessment and Mechanism Elucidation of Gelatinous Chinese Medicines

**DOI:** 10.3390/molecules31213812

**Published:** 2026-10-22

**Authors:** Lixin Zhou, Jianxiao Zheng, Ying Li, Kejing Niu, Fuxin Sun, Xin Chai, Yuefei Wang, Huijuan Yu

**Affiliations:** 1 State Key Laboratory of Chinese Medicine Modernization, Institute of Traditional Chinese Medicine, Tianjin University of Traditional Chinese Medicine, Tianjin 301617, China; zhoulixin2623@163.com (L.Z.); z13012621716@163.com (J.Z.); 18303616520@163.com (Y.L.); 18317357226@163.com (K.N.); fuxinsun0801@163.com (F.S.); chaix0622@tjutcm.edu.cn (X.C.); 2 Haihe Laboratory of Modern Chinese Medicine, Tianjin 301617, China

**Keywords:** gelatinous Chinese medicines, amino acids, nuclear magnetic resonance, chromatography techniques, quality evaluation

## Abstract

Gelatinous Chinese medicines (GCMs), such as Ejiao, are animal-derived solid preparations traditionally used for blood and qi deficiency. With growing market demand, adulteration has become a critical quality concern. This study established a multimodal quality assessment system for GCMs. Using ^1^H NMR, we characterized the acid hydrolysates of GCMs and identified 17 components, including 16 total hydrolysable amino acids and taurine, revealing compositional similarity across seven GCMs. Stability studies under acid hydrolysis were conducted on 19 amino acids, all of which remained stable except methionin, cystine, and tryptophan, leading to the selection of the remaining 16 amino acids as target analytes. A pre-column derivatization–HPLC method was validated for the quantification of 16 total hydrolysable amino acids, showing excellent linearity (r^2^ > 0.999), high precision (RSD < 2.71%), and satisfactory recoveries (90.74–109.3%). Content determination of 65 batches revealed batch-to-batch variations in total hydrolysable amino acid profiles. Combined with chemometrics, the method successfully discriminated dermal, horn, bone, and shell glues, and further enabled subcategory differentiation. Network pharmacology analysis suggested that these amino acid components may interact with key targets (e.g., GRM5, GABRA1, GABBR2) and pathways, primarily neuroactive ligand–receptor interaction. Collectively, this integrated analytical and network-based strategy not only supports chemical authentication but also provides insights into the potential molecular basis of the traditionally described restorative effects of GCMs, offering a scientific foundation for their modernization and international recognition.

## 1. Introduction

Gelatinous Chinese medicines (GCMs), such as Colla Corii Asini (Ejiao), are documented in the “*Recipes for Fifty-Two Ailments*”, China’s oldest known medical formulary, evidencing a history of application spanning over 2000 years [1]. GCMs are renowned for their actions in enriching blood, nourishing yin, moistening dryness, and arresting bleeding, which accounts for their extensive use in clinical treatment and healthcare maintenance [2,3,4]. GCMs are solid preparations obtained by aqueous decoction of collagen-rich animal tissues such as hides, horns, shells, and bones, followed by concentration and drying of the gelatinous extract [5]. GCMs are broadly classified by their zoological origin and medicinal parts into four categories: dermal glues (e.g., Colla Corii Asini, CCA; Colla Corii Bovis, CCB; Colla Corii Suilli, CCS), horn glues (e.g., Colla Cornus Cervi, CCC), bone glues (e.g., Colla Ossis Cervi, COC), and shell glues (e.g., Colla Carapacis et Plastri Testudinis, CCPT; Colla Carapacis Trionychis, CCT). GCMs are composed of animal proteins, peptides, amino acids, and a diverse range of trace elements [6,7,8,9]. Modern pharmacological studies have revealed that GCMs possess a range of activities, including hematopoietic effects, immunomodulatory properties, and the ability to alleviate hyperlipidemia, as well as anti-osteoporotic, anti-inflammatory, and analgesic actions [2,10,11,12]. The growing popularity of GCMs, particularly CCA (Ejiao), due their perceived efficacy and nutraceutical value, has contributed to a supply–demand crisis [13]. CCA is a solid glue crafted from the decoction and concentration of dried or fresh hides of the donkey (*Equus asinus* L.). It has been esteemed through the ages as a “superior tonic” for nourishment and a “sacred remedy” for replenishing blood, reflecting its long history in traditional Chinese medicine [14]. Rapid expansion of the health industry has further stimulated demand for GCMs. However, the raw materials for CCA, CCC, and COC, are inherently limited because donkeys or deer are not conventionally bred as livestock. This imbalance between supply and demand has promoted the circulation of counterfeit and adulterated products, which often involve substitution with hides from horses, cattle, pigs, sheep, or even industrial leather. Such practices pose potential risks to public health and seriously compromise the quality of authentic GCMs [15]. Therefore, establishing a scientific, efficient, and multi-dimensional quality evaluation system for GCMs has become an urgent requirement to ensure clinical efficacy and drug safety, and also represents an important issue for promoting the standardization and modernization of GCMs.

GCMs are primarily composed of proteins and their degradation products, amino acids, sugar substances, volatile substances, and small quantities of trace elements [5]. In its latest edition (2025), the Chinese Pharmacopoeia establishes quality standards for only three gelatinous medicines (CCA, CCC, and CCPT), which include the identification of characteristic peptides and the quantitation of four amino acids (glycine, alanine, hydroxyproline, and proline) [16]. Specifically, the content of these total hydrolysable amino acids is determined using hydrochloric acid hydrolysis followed by pre-column derivatization with phenyl isothiocyanate and high-performance liquid chromatography (HPLC). An HPLC-ELSD method was established to determine the hydrolyzed hydroxyproline content in CCPT, with a linear range of 0.1–1.0 µg/mL [17]. A liquid chromatography–mass spectrometry (LC-MS/MS) method has been developed for accurate quantification of gelatin peptide markers by utilizing the enhanced reaction induced by amino acid residues including glycine, alanine, and valine [6]. GC-MS was used to identify 16 characteristic fatty acids in CCB for origin tracing [18]. Existing studies have verified that near-infrared (NIR) spectroscopy combined with diverse chemometric algorithms can achieve accurate manufacturer identification and storage time prediction for Dong’e Ejiao with low misclassification rates [10]. In addition, a method integrating ultra-performance liquid chromatography–quadrupole time-of-flight mass spectrometry (UPLC/Q-TOF-MS) with principal component analysis (PCA) was established to differentiate CCA, CCB, CCS, CCPT, and CCC [19]. DNA barcoding has been employed to discriminate GCMs from different animal origins, such as distinguishing donkey-derived from horse-derived products [20]. Advanced computational algorithms have also been widely applied in drug discovery and target mining [21,22]. Currently, network pharmacology investigations of GCMs remain limited to compound formulations (e.g., Fufang E’jiao Jiang) or single-species studies, such as CCPT against perimenopausal insomnia [23,24,25].

Collectively, these studies have demonstrated various analytical platforms for the qualitative and quantitative characterization of GCMs. Despite these advances, most approaches remain isolated, focusing on chemical markers or species authentication without linking them to biological function. Moreover, the systematic relationship between total hydrolysable amino acid composition and restorative activity has yet to be explored. Herein, we propose an integrated strategy combining ^1^H NMR, HPLC, chemometrics, and network pharmacology to achieve comprehensive profiling, accurate classification, and simultaneous elucidation of key targets and pathways. This multimodal framework unifies quality control with mechanistic interpretation, offering a holistic foundation for the modernization of GCMs.

## 2. Results and Discussion

### 2.1. Analysis of Acid Hydrolysis Components of GCMs by ^1^H NMR Spectroscopy

In the ^1^H NMR spectrum of the acid-hydrolyzed CCA sample, proton signal peaks were distributed within the chemical shift range of *δ* 0.8–9.0 (Figure S1). Through comparative analysis with reference standards, a total of 17 compounds were identified (Figure S2), including 16 amino acids and one *β*-amino sulfonic acid, taurine. The assignment of ^1^H NMR chemical shifts in all 17 compounds in both the reference standards and the GCM hydrolysates are summarized in Table S2, allowing a direct peak-by-peak comparison.

The ^1^H NMR spectra of GCMs (including CCA, CCB, CCC, CCPT, CCT, COC, CCS, and EG) test solutions exhibited numerous and overlapping signals (Figure S3), indicating of the complex chemical composition of GCMs after acid hydrolysis. Notably, the ^1^H NMR spectra of GCM test solutions were relatively similar, with only subtle differences observed in the chemical shift range of 3.8–4.9 ppm. This might be primarily attributed to the peptide bond breakage caused by acid hydrolysis, which generated total hydrolysable amino acids. The α-hydrogen proton signals of these total hydrolysable amino acids typically appear in the range of 3.8–4.9 ppm, thereby accounting for the minor spectral differences in this specific region. These spectral differences likely reflect variations in the relative abundance of individual amino acids among different GCM types. In addition to the amino acids, taurine was also identified in the GCM hydrolysates. Taurine, a *β*-amino sulfonic acid, is a sulfur-containing non-proteinogenic amino acid widely distributed in animals. Because it lacks a carboxyl group and is not a standard amino acid, it was counted separately from the 16 amino acids. Therefore, the subsequent condition optimization and quantitative determination were performed only for the 16 amino acids. In this study, all amino acid contents were determined after acid hydrolysis and are therefore expressed as total hydrolysable amino acids. These findings suggest that while ^1^H NMR provides a holistic chemical fingerprint of GCM hydrolysates, its resolution for discriminating closely related varieties is limited without complementary quantitative approaches.

### 2.2. Stability of Amino Acids Under Acid Hydrolysis Conditions

**Figure 1:**
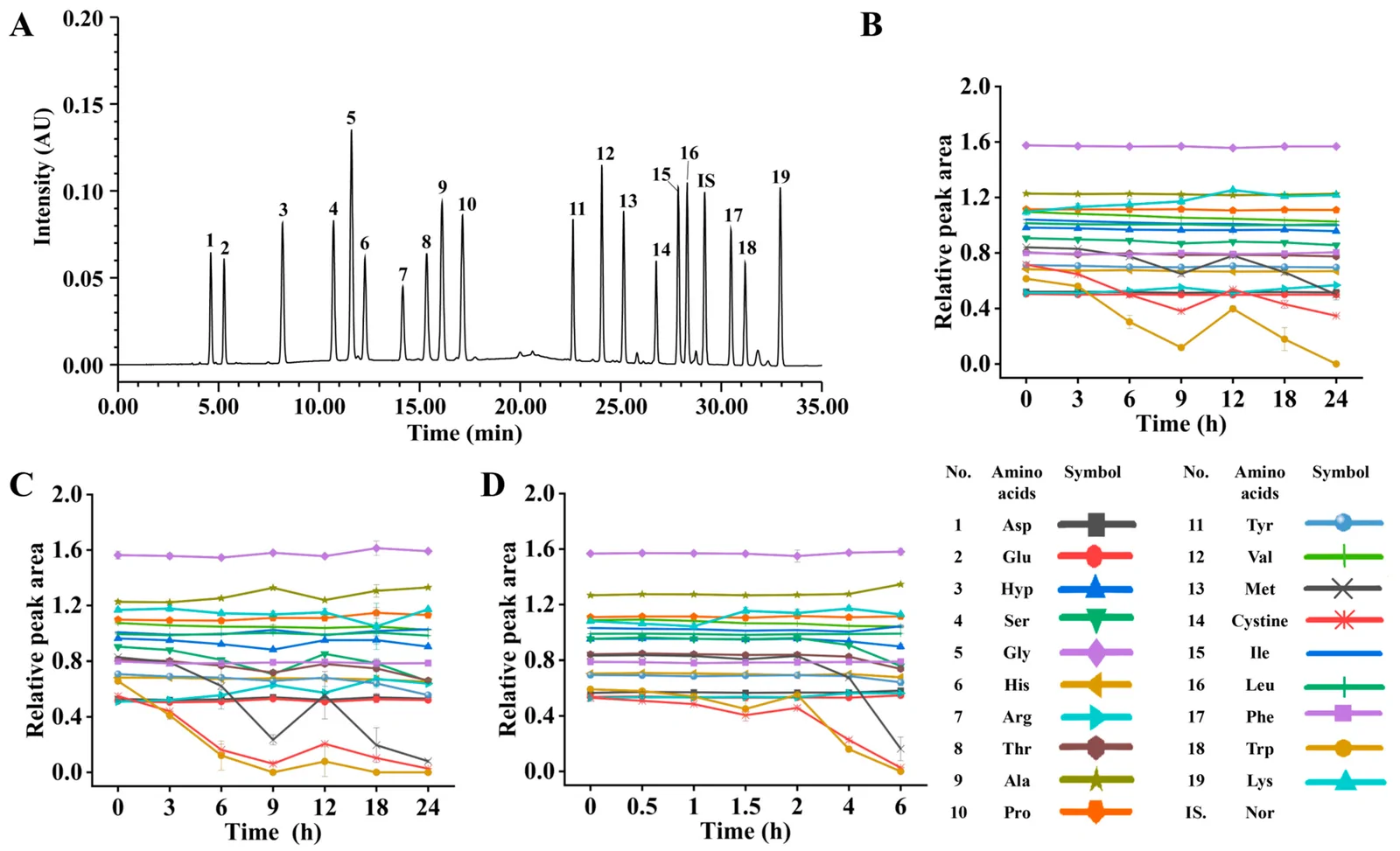
HPLC chromatograms of 19 amino acids (**A**) and the stability of the focused on amino acid under acid hydrolysis conditions at 110 °C (**B**), 130 °C (**C**), and 150 °C (**D**) (*n* = 3, x ± SD).

Protein hydrolysis is typically performed in 6 mol/L hydrochloric acid at temperatures ranging from 110 °C to 150 °C. Lower temperatures require longer hydrolysis times, whereas higher temperatures shorten the required time. Therefore, to accurately determine the content of hydrolyzed amino acids in GCMs, it is necessary to investigate the stability of each amino acid under acid hydrolysis conditions. In this study, using norleucine (Nor) as the internal standard (IS), the stability of 19 amino acids (Asp, Glu, Hyp, Ser, Gly, His, Arg, Thr, Ala, Pro, Tyr, Val, Ile, Leu, Phe, Lys, Met, cystine, and Trp) under hydrochloric acid hydrolysis at 110 °C, 130 °C, and 150 °C was investigated. As shown in Figure 1A, the 20 analytes (including IS) were well separated within 35 min.

The peak area of IS remained relatively stable (Figure S4). The relative peak area of each amino acid was calculated using Nor as the internal standard (Figure 1B–D). Two sulfur-containing amino acids (methionine and cystine) exhibited poor stability under hydrochloric acid hydrolysis at all three temperatures. Methionine is prone to oxidation, whereas cystine contains a labile disulfide bond that may undergo cleavage or degradation under strong acid conditions. This issue can be mitigated by adding an inert gas (such as nitrogen) or employing vacuum-based hydrolysis [26]. Tryptophan is completely degraded under acid hydrolysis conditions and typically requires separate determination by alkaline hydrolysis [27,28,29]. These findings underscore the necessity of excluding these three amino acids from routine acid hydrolysis-based quantification and highlight the importance of employing alternative methods when their determination is required. The remaining 16 amino acids (Asp, Glu, Hyp, Ser, Gly, His, Arg, Thr, Ala, Pro, Tyr, Val, Ile, Leu, Phe, and Lys) exhibited satisfactory stability under the tested conditions, confirming the reliability of the established hydrolysis protocol for routine analysis.

### 2.3. Optimization of Acid Hydrolysis and Derivatization Conditions for Total Hydrolysable Amino Acid Analysis in CCA

In this study, 16 amino acids were used as target analytes, and the total chromatographic peak area per unit mass (A/mg) was employed to systematically investigate the effects of acid hydrolysis conditions and derivatization conditions on the amino acid analysis of CCA (Table S3). For hydrolysis conditions, the effects of hydrolysis temperature (110, 130, and 150 °C) and hydrolysis time (1, 2, 3, 4, and 5 h) on the hydrolysis efficiency of amino acids were examined. For derivatization conditions, the effects of derivatization reagent concentration (0.1, 0.2, 0.3, 0.4, and 0.5 mol/L), derivatization temperature (25, 35, 45, and 55 °C), and derivatization time (20, 40, 60, 80, and 100 min) on the derivatization efficiency of amino acids were investigated.

**Figure 2:**
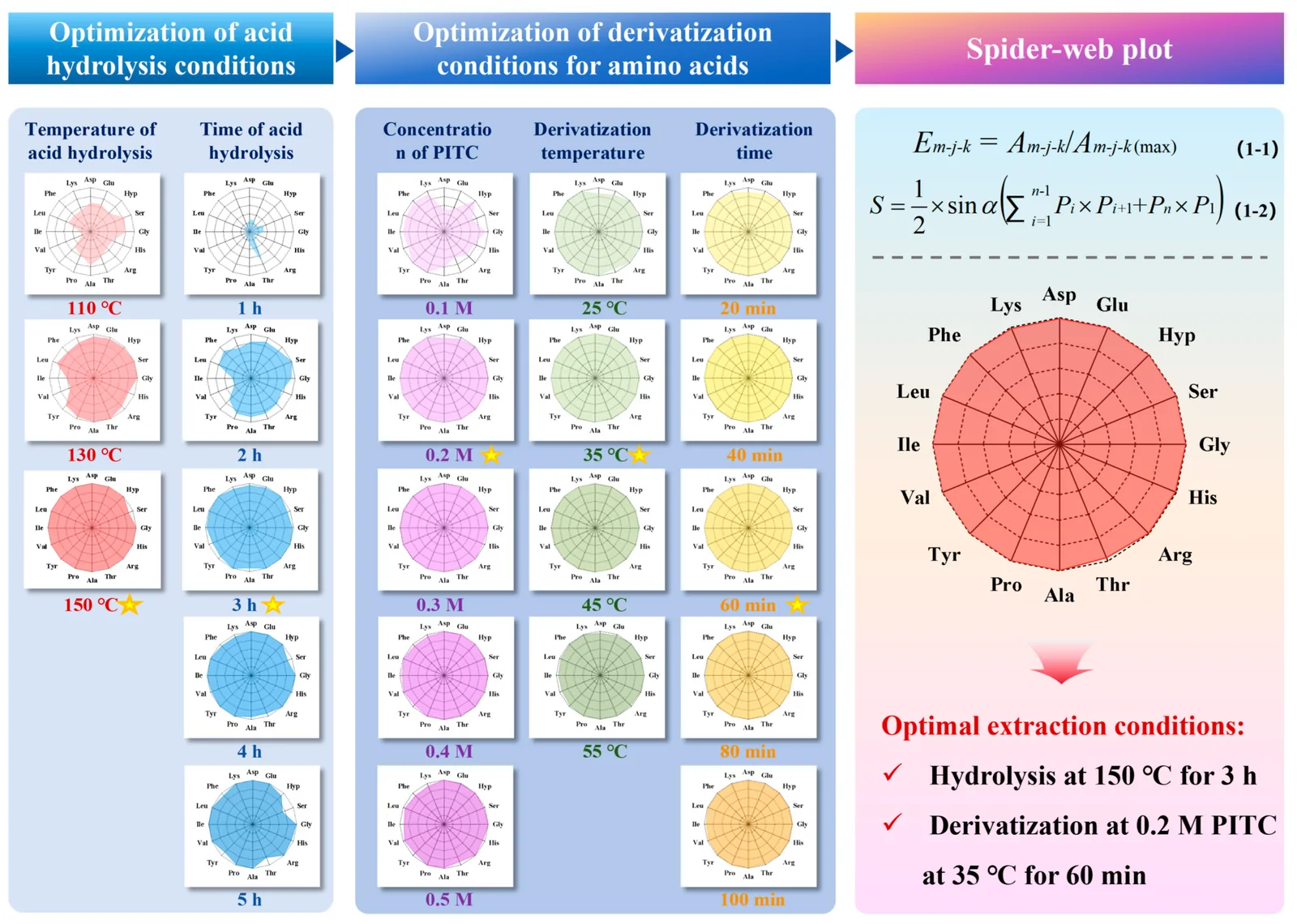
Application of a spider-web plot to the optimization of sample preparation conditions (★ indicates the optimal condition).

To visually demonstrate the optimization process of the preparation conditions for CCA test solutions, a spider-web plot was adopted to comprehensively analyze the effects of different acid hydrolysis and derivatization conditions on the extraction and derivatization efficiencies of amino acids from CCA. The unit-mass peak areas (A/mg) of 16 amino acids (Asp, Glu, Hyp, Ser, Gly, His, Arg, Thr, Ala, Pro, Tyr, Val, Ile, Leu, Phe, and Lys) obtained under different hydrolysis temperatures, hydrolysis times, derivatization reagent concentrations, derivatization temperatures, and derivatization times were designated as A_m-j-k_, where m represents the different hydrolysis or derivatization conditions, j represents the level of each parameter (e.g., 110, 130, and 150 °C for temperature), and k represents the individual amino acids. A_m-j-k(max)_ was defined as the maximum peak area of each amino acid. The normalized results E_m-j-k_ for each amino acid were calculated according to Equation (1-1) (Figure 2), and were presented in Table S4. E_m-j-k_ was then used to construct the spider-web plot pattern (P_i_) across different dimensions in Equation (1-2). The spider-web plot area (S) was calculated according to Equation (1-2), where α is the angle between two adjacent dimensions, i.e., α = 360°/*n* (*n* = 16). As shown in Figure 2, a larger shaded area in the spider-web plot indicates conditions closer to the optimal hydrolysis and derivatization conditions (Table S4).

Based on the single-factor optimization results, the optimal hydrolysis and derivatization conditions were selected as follows. Among the three temperatures tested (110, 130, and 150 °C), the total peak area of the 16 target amino acids was highest at 150 °C. At 150 °C, the total peak area increased from 1 h to 3 h and reached a maximum at 3 h, whereas prolonging the hydrolysis to 4 and 5 h did not further increase the peak area. These results indicate that 3 h at 150 °C was sufficient to achieve maximal measured release of the target amino acids. This temperature is also consistent with the 2025 edition of the Chinese Pharmacopoeia, which specifies 150 °C for the acid hydrolysis of Ejiao prior to amino acid determination. For derivatization, the highest total peak area was obtained at a PITC concentration of 0.2 mol/L, a derivatization temperature of 35 °C, and a derivatization time of 60 min, and no further increase was observed beyond these values. Therefore, the optimized parameters were as follows: acid hydrolysis temperature of 150 °C, acid hydrolysis time of 3 h, PITC concentration of 0.2 mol/L, derivatization temperature of 35 °C, and derivatization time of 60 min. It should be noted that the single-factor optimization combined with spider-web evaluation mainly reflects the effect of each parameter; statistically designed optimization strategies such as Box–Behnken design or response surface methodology can be considered in future studies.

In summary, the optimal hydrolysis and derivatization conditions were successfully established via spider-web plot-based multi-parameter optimization, providing a reliable and efficient sample preparation protocol for the total hydrolysable amino acid analysis of GCMs.

### 2.4. Methodological Validation for the Determination of Amino Acid Content in CCA

To identify total hydrolysable amino acids in different GCMs by HPLC, method validation was first performed using CCA as the model sample. Comprehensive method validation was performed in terms of linearity, limit of detection (LOD), limit of quantitation (LOQ), precision (intra-day and inter-day), repeatability, stability, and spiked recovery. As presented in Table S5, the calibration curves for the 16 amino acids were established, with correlation coefficients (r^2^) all exceeding 0.999, indicating excellent linearity within the respective concentration ranges tested. The LOD and LOQ ranged from 0.0085 to 0.1312 µg/mL and from 0.0254 to 0.3935 µg/mL, respectively, demonstrating the high sensitivity of the method. The RSD values for instrumental repeatability and instrumental inter-day precision were less than 0.41% and 2.71%, respectively. The RSD for independent sample-preparation repeatability and stability was below 2.92%, and the RSD for stability was below 1.09%. The mean recoveries ranged from 90.74% to 109.3%, with RSD values below 4.21%. Collectively, these validation results demonstrated that the established HPLC method was reliable, sensitive, and reproducible, and therefore suitable for the quantitative determination of amino acids in GCMs.

### 2.5. Determination of Amino Acid Content and Chemometric Analysis of Multi-Batch GCMs

Moisture content was determined for 65 batches of GCMs according to Section 3.9 (Table S6). The results showed that the moisture levels in all tested batches were below the limits specified by the 2025 edition of the Chinese Pharmacopoeia and relevant national standards (CCA, CCB, CCC, CCPT, CCT, COC, and CCS ≤ 15%; EG ≤ 14%), confirming that all batches met the regulatory requirements.

Amino acid contents in 65 batches of GCMs (calculated on a dry weight basis after moisture correction) are presented in Table S7. CCA was selected as a representative sample for detailed analysis. The results showed that Hyp content, a characteristic amino acid of collagen, ranged from 84.66 to 111.3 mg/g (RSD 7.63%), and all batches met the Pharmacopoeia requirement (≥8.0%, i.e., ≥80 mg/g). The Pro content ranged from 104.0 to 120.4 mg/g (RSD 4.86%), and all batches also met the pharmacopoeia requirement (≥10.0%, i.e., ≥100 mg/g). However, the Gly content ranged from 168.9 to 198.0 mg/g (RSD 5.08%), with seven batches (CCA10–13, CCA15–16, and CCA23) falling below the pharmacopoeia limit of 18.0% (180 mg/g). The Ala content ranged from 68.86 to 89.62 mg/g (RSD 6.69%), with one batch (CCA12) below the pharmacopoeia limit of 7.0% (70 mg/g).

In addition, the total content of six essential amino acids (EAAs; Thr, Val, Ile, Leu, Phe, and Lys) ranged from 105.9 to 145.4 mg/g (RSD 8.33%), with the highest value in CCA9. The total content of nine non-essential medicinal amino acids (NEAAs; Asp, Glu, Hyp, Ser, Gly, His, Arg, Pro, and Val) ranged from 568.26 to 687.76 mg/g (RSD 5.97%), with the highest value in CCA22. The total content of six umami amino acids (Asp, Glu, Gly, Ala, Tyr, and Phe) ranged from 379.2 to 456.8 mg/g (RSD 5.73%, highest value in CCA9); that of six sweet amino acids ranged from 392.2 to 461.2 mg/g (RSD 5.33%, highest value in CCA22); and that of two aromatic amino acids ranged from 21.00 to 26.11 mg/g (RSD 7.57%, highest value in CCA22).

The amino acid contents of the other seven types of GCMs (CCB, CCC, CCPT, CCT, COC, CCS, and EG) are summarized in Table S8. Overall, considerable variations in amino acid contents were observed among different GCMs varieties, with CCPT showing the greatest variability (RSD of Hyp up to 13.13%) and CCT showing relatively smaller variability (RSD of Hyp at 2.99%). The total content ranges of EAAs, NEAAs, umami amino acids, sweet amino acids, and aromatic amino acids for each variety are also provided in Table S8.

**Figure 3:**
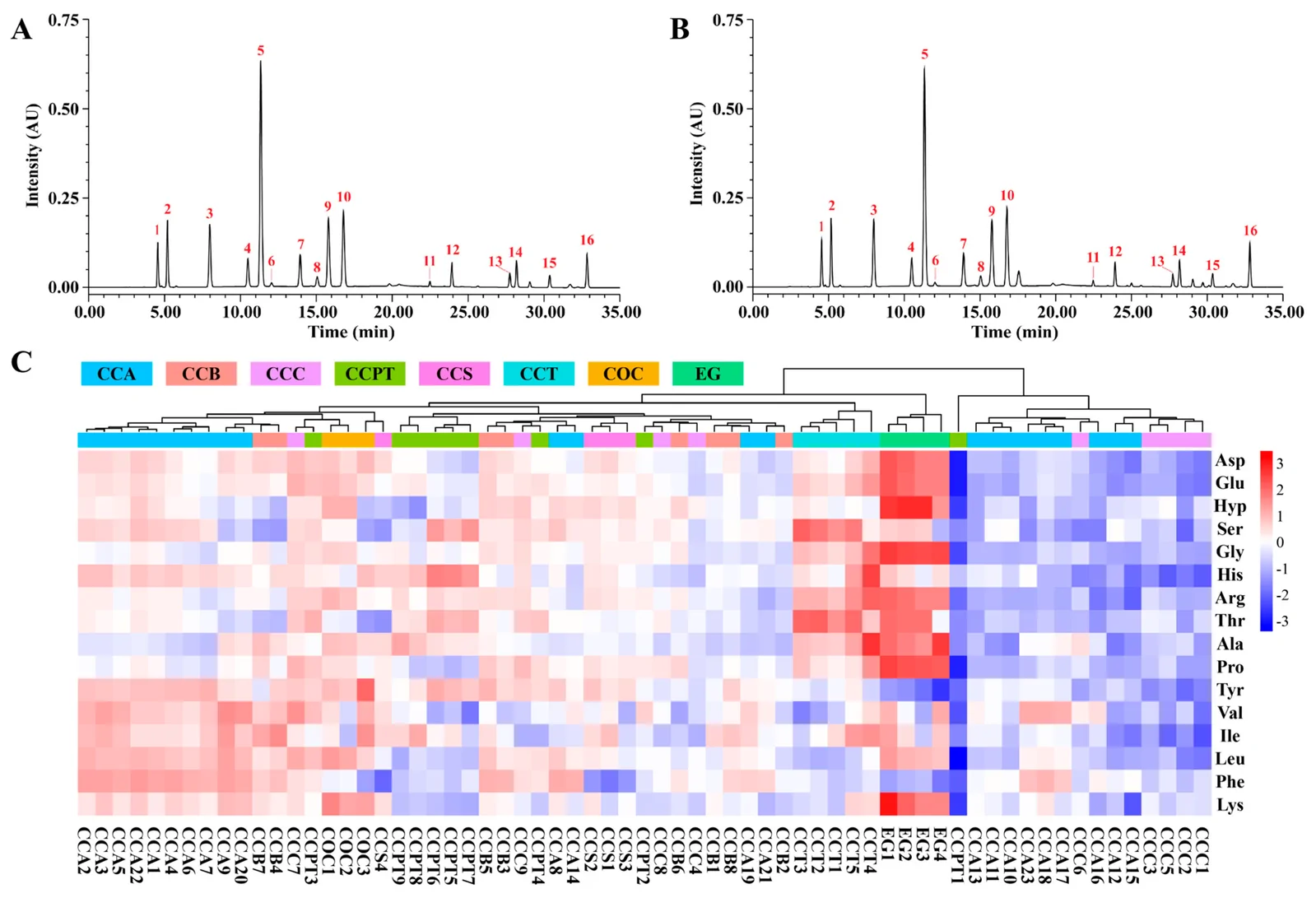
HPLC chromatograms of (**A**) mixed reference solution and **(B**) CCA sample solution, and (**C**) cluster heat map of 16 amino acid components in 65 batches of GCMs. Peak identities: 1. Asp; 2. Glu; 3. Hyp; 4. Ser; 5. Gly; 6. His; 7. Arg; 8. Thr; 9. Ala; 10. Pro; 11. Tyr; 12. Val; 13. Ile; 14. Leu; 15. Phe; 16. Lys.

Cluster analysis is an unsupervised learning method that aims to partition samples or variables into distinct groups based on data similarity, such that objects within the same group are as similar as possible, while objects in different groups are as dissimilar as possible. The contents of 16 total hydrolysable amino acids in 65 batches of GCMs were normalized for cluster analysis. As shown in Figure 3C, the color scale from red to blue represents the contents from high to low, indicating considerable variations in amino acid contents among different batches of GCMs. The clustering results showed that only CCT and EG could be grouped into distinct clusters, whereas the other GCM varieties exhibited relatively scattered clustering patterns. Therefore, cluster analysis alone was insufficient to clearly differentiate the various types of GCMs, and further chemometric methods were required.

**Figure 4:**
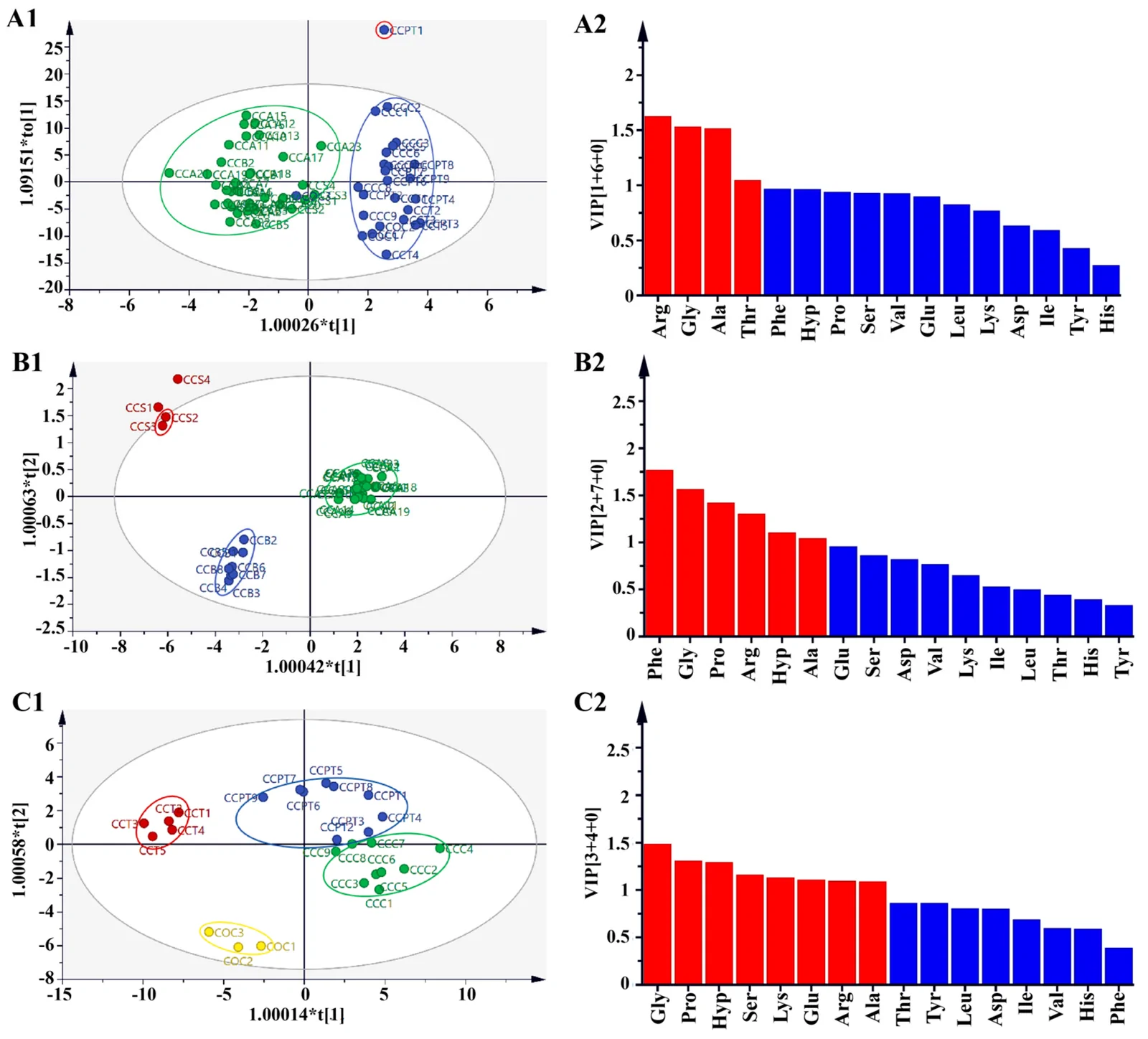
OPLS-DA score plots and VIP analysis of GCMs. (**A1**) Score plot for distinguishing skin-derived gels (CCA, CCB, CCS) from shell/horn/bone-derived gels (CCPT, CCT, CCC, COC). (**A2**) VIP values of differential amino acids between skin-derived gels and shell/horn/bone-derived gels. (**B1**) Score plot for distinguishing among CCA, CCB, and CCS. (**B2**) VIP values of differential amino acids among CCA, CCB, and CCS. (**C1**) Score plot for discrimination among CCC, CCPT, CCT, and COC. (**C2**) VIP values of differential amino acids among CCC, CCPT, CCT, and COC.

To more effectively discriminate among different types of GCMs, the seven GCM varieties (excluding EG) were divided into two major categories: skin-derived gels (including CCA, CCB, and CCS) and shell/horn/bone-derived gels (including CCPT, CCT, CCC, and COC). OPLS-DA was then performed based on this classification. As shown in Figure 4(A1), the green dots represent skin-derived gel samples, and the blue dots represent shell/horn/bone-derived gel samples. Within the 95% confidence region, the two categories were clearly separated, with only one sample (COC3) misclassified into the skin-derived gel group. Notably, sample CCPT1 exhibited significantly lower amino acid contents (Figure 3C) than the other batches and fell outside the 95% confidence region; and was therefore labeled as an outlier (red circle). The OPLS-DA model yielded cumulative explanatory ability R^2^X and R^2^Y of 0.975 and 0.842, respectively, and a predictive parameter Q^2^ of 0.751, all exceeding 0.5, indicating that the established model was stable and reliable. To avoid potential overfitting, a 200-permutation test was performed (Figure S6). The permutation test results for the skin-derived gel group [R^2^ = (0.0, 0.152), Q^2^ = (0.0, −0.443)] and for the shell/horn/bone-derived gel group [R^2^ = (0.0, 0.160), Q^2^ = (0.0, −0.432)] both indicated no overfitting, further confirming the robustness and credibility of the model. Variable importance in projection (VIP) is an important indicator that quantifies the contribution of each variable to model classification. Using VIP > 1 (marked in red in the figure) as the criterion for screening differential markers, four amino acids (Arg, Gly, Ala, and Thr) were identified as the key differential components discriminating skin-derived gels from shell/horn/bone-derived gels (Figure 4(A2)). Moreover, the average contents of these four amino acids were higher in the shell/horn/bone-derived gels than in the skin-derived gels. This compositional difference likely reflects the distinct protein sources of these materials: skin-derived gels are predominantly type I collagen, while shell, horn, and bone tissues contain keratin and sclerotin with higher Arg and Thr proportions [30,31].

OPLS-DA was also applied to further distinguish the three skin-derived gels (CCA, CCB, and CCS). As shown in Figure 4(B1), the three types were well separated within the 95% confidence region. The permutation test (Figure S6) confirmed the reliability of the model (Q^2^ intercepts: −0.841 for CCA, −0.849 for CCB, and −0.887 for CCS). Based on VIP > 1, six amino acids (Phe, Gly, Pro, Arg, Hyp, and Ala) were identified as differential markers (Figure 4(B2)). Among the six differential markers, Gly, Pro, and Hyp are characteristic amino acids of collagen, with Hyp being a unique post-translational modification product found almost exclusively in collagen [32,33]. Their content order (CCS > CCB > CCA) reflects differences in collagen composition and degree of hydroxylation among species. Porcine skin (CCS) and bovine skin (CCB) typically contain higher levels of imino acids (Pro and Hyp) than donkey skin (CCA). Arg, a basic amino acid involved in immune regulation and wound healing, was also highest in CCS, consistent with the traditional use of porcine-derived gelatin in tissue repair [34,35]. Notably, Phe showed an opposite trend (CCA > CCB > CCS), suggesting that aromatic amino acid content varies inversely with imino acid abundance across species. Collectively, these six amino acids not only enable species-level discrimination but also provide a chemical basis for differentiating the functional properties of skin-derived GCMs.

Similarly, OPLS-DA successfully distinguished the four shell/horn/bone-derived gels (CCC, CCPT, CCT, and COC) (Figure 4(C1)), with Q^2^ intercepts from permutation tests all below zero (Figure S6). Eight amino acids (Gly, Pro, Hyp, Ser, Lys, Glu, Arg, and Ala) were identified as key differential components (VIP > 1, Figure 4(C2)). Gly, Pro, and Hyp, collagen-characteristic amino acids, were higher, as expected in COC (bone-derived), as bone matrix is predominantly type I collagen. In contrast, CCPT and CCT (shell-derived) are rich in insoluble sclerotin and calcium-binding proteins, which may account for their distinct profiles of Ser, Lys, and Glu-amino acids commonly associated with calcified tissues [36]. CCC (horn-derived), which contains substantial keratin, exhibited higher levels of Arg and Ala, consistent with the known abundance of these amino acids in keratinous proteins. Ser and Lys are also involved in post-translational crosslinking, which is particularly relevant to the structural stability of shell and horn tissues [37]. These compositional variations not only support the robust classification observed but also suggest that different amino acid markers may be associated with specific material properties, such as mechanical strength (shell/bone) or elasticity (horn)—thereby providing a chemical rationale for the distinct applications of these GCMs in traditional medicine.

In summary, these findings demonstrate that OPLS-DA combined with total hydrolysable amino acid profiling enables stepwise and accurate discrimination of GCMs across different tissue origins and species. The identified VIP markers-particularly collagen-associated (Gly, Pro, and Hyp), basic (Arg, Lys), and hydroxyl/amide-related (Ser, Glu, and Ala) amino acids-not only serve as reliable chemical fingerprints for quality control but also reflect the inherent biochemical differences in protein composition among raw materials, offering a mechanistic foundation for the rational use and quality evaluation of diverse GCM varieties.

### 2.6. Network Pharmacological Study on the Tonic Activity of Amino Acid Components in GCMs

#### 2.6.1. Amino Acid-Restorative Activity Target Network

**Figure 5:**
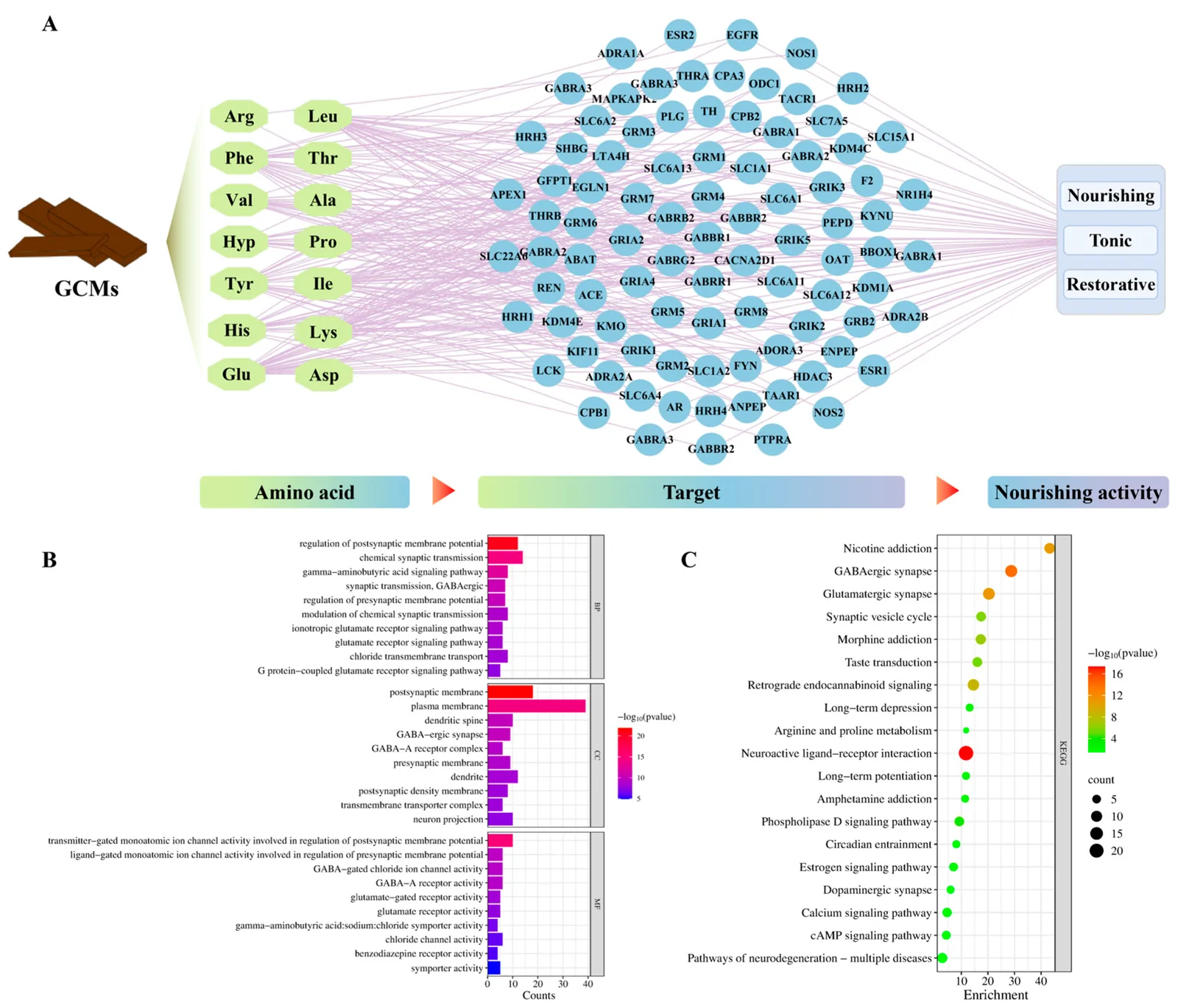
Network diagram of “amino acid composition–target–activity” (**A**). Histogram of GO functional enrichment analysis (BP, CC, MF) (**B**). Bubble diagram of KEGG pathway enrichment analysis (**C**).

Network pharmacology provides a systematic strategy to dissect the multitarget regulatory mechanisms of natural products [38]. Among the 16 target amino acids, Gly and Ser were excluded because no predicted targets with Probability* > 0 were available, leaving 14 amino acids for network analysis. A total of 86 targets corresponding to 14 amino acids were obtained after searching the SwissTargetPrediction database after integration and removal of duplicates (Table S9). A total of 2577 gene targets associated with the restorative activity of GCMs were retrieved from the GeneCards database. Subsequently, the targets of the amino acid components and the GCM restorative targets were intersected using the Venny online tool, yielding 47 overlapping targets (Table S10). A “component–target–activity” network was constructed using Cytoscape 3.7.1 software (Figure 5A). The node degree was used as the core evaluation metric, which is positively correlated with the number of edges connected to a node. A higher node degree indicates that the component or target plays a more critical role in network regulation. Six amino acid components with a degree > 10 (Table S11), namely Leu, Ile, Glu, Lys, Phe, and Val, were identified as key nodes due to their extensive interactions with restorative targets.

#### 2.6.2. PPI Network Construction and Key Targets Analysis

The 47 overlapping target genes of the amino acid-restorative activity were imported into the STRING database to construct a PPI network with a confidence score ≥ 0.4. The network comprised 46 nodes and 225 interactions. The above network data were imported into Cytoscape 3.7.1 software, and the top 20 targets (including 149 interactions) screened by the Maximum Clique Centrality (MCC) algorithm in the CytoHubba plugin were used to construct a PPI network (Figure S7). In the network, a larger node area and darker color indicate a higher degree, and thus greater importance of the node in the network. The targets, including GRM5, GABRA1, GRIA2, GRIA1, GRIA4, GRM7, GABBR2, GABRG2, GRIK5, GABRB2, GABRA3, GABRA2, GABBR1, GRIK1, GRM4, GRM1, GRIK2, SLC6A1, and SLC1A2, all had degree > 10. GRM1/4/5/7 encode the metabotropic glutamate receptor; GRIA1/2/4 encode the ionotropic AMPA receptor; GRIK1/2/5 encode the ionotropic kainate receptor; GABRA1/2/3, GABRB2, and GABRG2 encode the GABAA receptor; GABBR1/2 encode the GABAB receptor; SLC1A2 encodes the glutamate transporter; and SLC6A1 encodes the GABA transporter. These core targets synergistically regulate the glutamate-GABA neurotransmitter system, which is closely related to the regulation of neural function, energy metabolism, and immune homeostasis, which are key biological processes underlying the restorative effect of GCMs. Collectively, they constitute the regulatory network underlying the restorative activity of amino acids.

#### 2.6.3. GO Functional Enrichment Analysis and KEGG Pathway Enrichment Analysis Results

Subsequently, the overlapping targets were imported into the DAVID database for GO functional and KEGG pathway enrichment analyses. GO analysis revealed 108 biological processes (BP), 45 cellular components (CC), and 56 molecular functions (MF) at *p* < 0.05. The top 10 terms in each category, ranked by *p*-value, were selected for visualization (Figure 5B). BP were mainly related to the regulation of postsynaptic membrane potential, chemical synaptic transmission, the gamma-aminobutyric acid (GABA) signaling pathway, and GABAergic synaptic transmission. CC were mainly related to the postsynaptic membrane, plasma membrane, dendritic spine, and GABAergic synapse. MF were mainly related to neurotransmitter-gated ion channel activity involved in the regulation of postsynaptic membrane potential, and ligand-gated ion channel activity involved in the regulation of presynaptic membrane potential.

A total of 23 pathways were obtained from KEGG pathway enrichment analysis, and the top 20 pathways were visualized as a bubble plot (Figure 5C). The results revealed that the restorative effects of amino acid components in GCMs mainly involve neuroactive ligand–receptor interaction, GABAergic synapse, glutamatergic synapse, nicotine addiction, and retrograde endocannabinoid signaling, among others. Among them, the most significant was the neuroactive ligand–receptor interaction pathway, which involves many plasma membrane receptors and ligands involved in intra- and extracellular signaling. Amino acid components mainly regulate this pathway by binding to targets such as GRM, GABBR, GABRA, GABRB, and GRIK.

Collectively, these network pharmacology analyses reveal that the restorative activity of GCMs is associated with six key amino acids (Leu, Ile, Glu, Lys, Phe, and Val), which act on 20 core targets. Overall, the network approach bridges chemical composition with biological function, offering a mechanistic foundation for the tonic effects of GCMs and guiding future quality-efficacy studies.

## 3. Materials and Methods

### 3.1. Reagents and Materials

LC grade acetonitrile was purchased from Sigma-Aldrich Inc. (St. Louis, MO, USA). Glacial acetic acid was acquired from Tianjin Huihang Chemical Technology Co., Ltd. (Tianjin, China). Analytical grade phenyl isothiocyanate (PITC) and triethylamine were purchased from Shanghai Aladdin Biochemical Technology Co., Ltd. (Shanghai, China). N-hexane (analytical grade) was obtained from Tianjin Concord Technology Co., Ltd. (Tianjin, China). Analytical grade hydrochloric acid was purchased from Tianjin Damao Chemical Reagent Factory (Tianjin, China). Reference standards including glycine (Gly, H04J10Z79020), L-proline (Pro, Z12O11H127142), L-hydroxyproline (Hyp, A30IB224213), L-alanine (Ala, H22N11Y131694), L-glutamate (Glu, J30S9R71489), L-arginine (Arg, H11M10Y82633), L-aspartic acid (Asp, S24A8I34463), L-lysine (Lys, S09O11I126602), L-leucine (Leu, BCBN3570V), L-serine (Ser, J01GB150454), L-valine (Val, S10M11I109402), L-phenylalanine (Phe, H02J11H117318), L-isoleucine (Ile, S20N11I131753), L-threonine (Thr, J24N8R48850), L-tyrosine (Tyr, J30M10R84464), L-histidine (His, Z18A11H121745), L-methionine (Met, H28M9H62331), L-cystine (H07N11Y130287), L-tryptophan (Trp, S07D7I26134), and L-norleucine (Nor, H05JS1) were acquired from Shanghai Yuanye Biotech Co., Ltd. (Shanghai, China). Taurine (111616-201002) was acquired from the National Institutes for Drug Control (Beijing, China). The purity of all standards was above 98%. Anhydrous sodium acetate (analytical reagent) was purchased from Tianjin Fengchuan Chemical Reagent Technology Co., Ltd. (Tianjin, China). The information of different batches of GCMs samples was shown in Table S1.

### 3.2. Sample Preparation for Reference Standard Stability Study

Twenty reference standards were accurately weighed and individually dissolved in 0.1 mol/L hydrochloric acid to prepare individual stock solutions. These stock solutions were subsequently combined to prepare a mixed standard solution with the following final concentrations: 100.1 µg/mL Gly, 100.8 µg/mL Pro, 99.90 µg/mL Hyp, 99.72 µg/mL Ala, 100.3 µg/mL Glu, 99.88 µg/mL Arg, 100.0 µg/mL Asp, 100.1 µg/mL Lys, 100.2 µg/mL Leu, 99.50 µg/mL Ser, 100.5 µg/mL Val, 100.1 µg/mL Phe, 100.3 µg/mL Ile, 100.1 µg/mL Thr, 100.3 µg/mL Met, 100.7 µg/mL Tyr, 100.3 µg/mL His, 79.52 µg/mL Cystine, 100.8 µg/mL Trp, and 100.1 µg/mL Nor (internal standard, IS).

A 2-mL aliquot of the mixed standard solution was transferred into a reaction vessel, followed by the addition of 2 mL of 6 mol/L hydrochloric acid, giving a final HCl concentration of approximately 3 mol/L. HCl was added to provide an acidic environment comparable to that of the sample hydrolysis procedure, although the amino acid standards themselves do not require hydrolysis. The hydrolysis reaction was respectively performed at 110 °C for 0, 3, 6, 9, 12, 18, and 24 h; at 130 °C for 0, 3, 6, 9, 12, 18, and 24 h; or at 150 °C for 0, 0.5, 1, 1.5, 2, 4, and 6 h. After cooling to room temperature, the reaction mixture was transferred to an evaporating dish. Following this, the reaction vessel was rinsed multiple times with a total of 5 mL of ultrapure water, and the washings were combined into the same dish. The combined solution was then evaporated to dryness in a 98 °C water bath and reconstituted with 1 mL 0.1 mol/L hydrochloric acid to obtain reference solution. The reference solution (200 μL) was treated sequentially with 100 μL of 0.1 mol/L PITC acetonitrile solution and 100 μL of 1 mol/L triethylamine acetonitrile solution. After thorough mixing, the solution was allowed to react at room temperature for 1 h to complete the derivatization. Following derivatization, the mixture was subjected to liquid–liquid extraction with 400 μL of n-hexane (vortexed thoroughly and then allowed to stand for 10 min), and the extraction was repeated twice. Take 100 μL of the lower extraction solution, dilute it with 900 μL of ultrapure water, and centrifuge at 12,700 rpm for 10 min to prepare the test solution for amino acid stability study.

### 3.3. Optimization of Acid Hydrolysis Conditions for CCA

CCA sample powder (passed through a No. 2 sieve) of approximately 25 mg was accurately weighed and placed into a 25 mL reaction vessel. Hydrochloric acid solution (6 mol/L, 10 mL) was added, and the mixture was ultrasonicated (500 W) for 10 min, then allowed to cool. For hydrolysis temperature optimization, the samples were reacted at 110, 130, and 150 °C for 3 h; for hydrolysis time optimization, the samples were reacted at 150 °C for 1, 2, 3, 4, and 5 h. After the reaction, the mixtures were cooled to room temperature, transferred to evaporating dishes, and washed with ultrapure water (10 mL) in portions. The washings were combined and evaporated to dryness at 98 °C. The residue was dissolved in 5 mL of 0.1 mol/L hydrochloric acid and mixed well. An aliquot (200 μL) was transferred to a centrifuge tube, and 100 μL each of 0.1 mol/L phenyl isothiocyanate in acetonitrile and 1 mol/L triethylamine in acetonitrile were added. The mixture was vortexed and allowed to stand at room temperature (25 °C) for 1 h for derivatization. Subsequently, 400 μL of n-hexane was added, vortexed, and allowed to stand for 10 min; this extraction was repeated twice. The lower layer (100 μL) was taken, diluted with 900 μL of 0.1 mol/L sodium acetate (pH 6.5 with glacial acetic acid)–acetonitrile solution (93:7, *v*:*v*), and mixed. After centrifugation at 12,700 rpm for 10 min, the supernatant was filtered through a 0.45 μm membrane, and the filtrate was collected as the test solution.

### 3.4. Optimization of Amino Acid Derivatization Conditions for CCA

CCA sample powder (passed through a No. 2 sieve) of approximately 25 mg was accurately weighed and placed into a 25 mL PTFE digestion vessel. Hydrochloric acid (6 mol/L, 10 mL) was added, and the mixture was ultrasonicated (500 W) for 10 min, then allowed to cool and reacted at 150 °C for 3 h. After cooling to room temperature, the mixture was transferred to an evaporating dish and washed with ultrapure water (10 mL) in portions. The washings were combined and evaporated to dryness at 98 °C. The residue was dissolved in 5 mL of 0.1 mol/L hydrochloric acid and mixed well. An aliquot (200 μL) was transferred to a centrifuge tube, and 100 μL of 1 mol/L triethylamine in acetonitrile was added. Derivatization was performed under the following conditions: for PITC concentration optimization, 100 μL of 0.1, 0.2, 0.3, 0.4, and 0.5 mol/L PITC in acetonitrile was added, and the reaction was carried out at 35 °C for 60 min; for temperature optimization, 100 μL of 0.2 mol/L PITC solution was added, and the reaction was carried out at 25, 35, 45, and 55 °C for 60 min; for time optimization, 100 μL of 0.2 mol/L PITC solution was added, and the reaction was carried out at 35 °C for 20, 40, 60, 80, and 100 min. After derivatization, 400 μL of n-hexane was added, vortexed, and allowed to stand for 10 min; this extraction was repeated twice. The lower layer (100 μL) was taken, diluted with 900 μL of 0.1 mol/L sodium acetate (pH 6.5 with glacial acetic acid)–acetonitrile solution (93:7, *v*:*v*), and mixed. After centrifugation at 12,700 rpm for 10 min, the supernatant was filtered through a 0.45 μm membrane, and the filtrate was collected as the test solution.

### 3.5. Preparation of Standard and Sample Solutions for Quantitative Analysis

#### 3.5.1. Preparation of Standard Solutions

Sixteen target amino acid reference standards (screened for satisfactory stability under acid-hydrolysis conditions) were accurately weighed and dissolved in hydrochloric acid (0.1 mol/L) to obtain stock solutions separately, which were used to prepare a mixed standard solution with final concentrations of 3.746 mg/mL Gly, 2.096 mg/mL Pro, 1.830 mg/mL Hyp, 1.511 mg/mL Ala, 1.684 mg/mL Glu, 1.511 mg/mL Arg, 0.9195 mg/mL Asp, 0.7660 mg/mL Lys, 0.5460 mg/mL Leu, 0.6288 mg/mL Ser, 0.4265 mg/mL Val, 0.3395 mg/mL Phe, 0.2925 mg/mL Ile, 0.2955 mg/mL Thr, 0.1399 mg/mL Tyr, and 0.1251 mg/mL His. The mixed standard solution was serially diluted with 0.1 mol/L hydrochloric acid solution by factors of 2, 4, 8, 16, 32, and 64 to obtain a series of working solutions at varying concentrations for establishing the calibration curves.

#### 3.5.2. Preparation of Acid-Hydrolyzed Sample Solutions of GCMs

Approximately 25 mg of each sample powder (CCA, CCB, CCC, CCPT, CCT, COC, and CCS), passed through a No. 2 sieve, was accurately weighed and transferred into a 25 mL reaction vessel liner. Subsequently, 10 mL of 6 mol/L hydrochloric acid was added, and the mixture was sonicated (500 W) for 10 min. After cooling, the hydrolysis was conducted at 150 °C for 3 h. After cooling to ambient temperature, the hydrolysate was transferred to an evaporating dish. The reaction vessel liner was rinsed several times with a total of 10 mL of water, and all rinsates were combined into the same dish. The combined liquid was then evaporated to dryness at 98 °C. The dried residue was subsequently dissolved in 5 mL of 0.1 mol/L hydrochloric acid solution and mixed thoroughly to obtain the final solution.

#### 3.5.3. Derivatization of Standard and Sample Solutions

The reference solution (“Section 3.5.1”) and sample solution (“Section 3.5.2”) were derivatized separately before analysis. A 200-μL aliquot of standard or sample solution was treated sequentially with 100 μL of 0.2 mol/L PITC acetonitrile solution and 100 μL of 1 mol/L triethylamine acetonitrile solution. After thorough mixing, the solution was allowed to react at 35 °C for 1 h to complete the derivatization. Following derivatization, the mixture was subjected to liquid–liquid extraction with 400 μL of n-hexane (vortexed thoroughly and then allowed to stand for 10 min), and the extraction was repeated twice. Take 100 μL of the lower extraction solution, dilute it with 900 μL of 0.1 mol/L sodium acetate (pH 6.5 with glacial acetic acid)–acetonitrile solution (93:7, *v*:*v*), and centrifuge at 12,700 rpm for 10 min to obtain the supernatant for analysis.

### 3.6. NMR Spectroscopic Analysis

Nuclear magnetic resonance (NMR) analysis was performed on a Bruker AVANCE III 600 spectrometer operating at 600 MHz for ^1^H NMR detection and equipped with an ultra-low-temperature probe. The dried residues obtained from acid-hydrolysis of GCMs (Section 3.5.2) were redissolved in D_2_O containing sodium 3-(trimethylsilyl)propionate-2,2,3,3-d_4_ (TSP-d_4_) as the internal standard (final concentration, 0.9961 mM). No additional pH adjustment or buffer was applied. After vortex mixing and centrifugation at 14,000 rpm for 15 min, 500 μL of supernatant was transferred to a 5 mm NMR tube for measurement. The sample temperature was precisely maintained at 298.0 K throughout the experiment. The ^1^H NMR spectra of the GCMs test solutions were acquired using the zgpr presaturation monopulse sequence for water suppression. Chemical shifts (*δ*) are reported in ppm relative to the internal standard, TSP-d_4_, and coupling constants (J) are reported in Hz. The amino acid reference standards were directly dissolved in D_2_O/TSP-d_4_ without acid addition. The amino acid signals in the GCM hydrolysates were assigned by comparing their chemical shifts and peak patterns with those of the reference standards. All GCM samples were prepared under identical conditions.

### 3.7. HPLC-PDA Conditions

An LC 20-AT system (Shimadzu Corporation, Kyoto, Japan) was used to perform the chromatographic analysis using an Ultimate^®^ Amino Acid column (4.6 × 250 mm, 5 µm) at 40 °C for the quantitative analysis. The mobile phase was composed of 0.1 mol/L sodium acetate solution (pH 6.5 with glacial acetic acid)–acetonitrile (93:7) (A) and water–acetonitrile (20:80) (B), and then implemented in the gradient elution as follows: 0–11 min, 0–7% B; 11–13.9 min, 7–12% B; 13.9–14 min, 12–15% B; 14–29 min, 15–24% B; 29–32 min, 24–70% B; and 32–35 min, 70–100% B. The flow rate was 1.0 mL/min, and the sample injection was 10 µL. The detection wavelength was set at 254 nm.

### 3.8. Methodological Validation

The analytical method established in this study was validated for linearity, limit of detection (LOD), limit of quantitation (LOQ), instrumental repeatability, instrumental inter-day precision, independent sample-preparation repeatability, stability, and spiked recovery. Calibration curves were constructed with the peak area as the ordinate (*y*-axis) and the corresponding concentrations (μg/mL) of the target compounds as the abscissa (*x*-axis). The LOQ and LOD of the tested compounds were determined by sequentially diluting the concentration of the standard solution until the signal-to-noise ratio (S/N) reached approximately 10 and 3, respectively. Instrumental repeatability, was evaluated by injecting the sample solution six times on the same day. Instrumental inter-day precision was evaluated by injecting the sample solution six times daily for three consecutive days (*n* = 6 per day). Independent sample-preparation repeatability was evaluated by independently preparing six sample solutions from the same batch of CCA, and each solution was injected once. Stability was investigated by injecting the same sample solution at 0, 2, 4, 6, 8, 10, 12, and 24 h after preparation. The spiked recovery test was performed by accurately adding 1 mL of the mixed amino acid standard solution, containing amounts equivalent to those present in the sample, to about 25 mg of CCA sample powder (*n* = 6). The spiked samples were processed according to the same procedure as described in Section 3.5.2, and each sample was injected twice. Recovery was calculated as (measured amount − original amount)/spiked amount × 100%.

### 3.9. Moisture Determination Conditions

According to the “Drying Loss Determination” (General Rule 0832, Method 2) specified in the Chinese Pharmacopoeia (2025 edition) (Commission 2025) [3,16], approximately 1 g of each powdered sample (CCA, CCC, CCPT, CCB, CCT, CCS, COC, and EG), which had been sieved through a No. 2 sieve, was precisely weighed into a pre-dried flat-bottomed weighing bottle. Following dissolution in 2 mL of water with heating, the sample was reweighed. The bottle was then uncapped and dried at a constant temperature of 105 °C for 5 h. Subsequently, the weighing bottle was capped and transferred to a desiccator for cooling to room temperature (30 min), followed by precise weighing. The above drying procedure was repeated: the uncapped bottle was dried at 105 °C for 1 h each time, then capped, cooled in the desiccator, and weighed. The moisture content of each sample was calculated based on the weight loss, and all determinations were performed in triplicate (*n* = 3).

### 3.10. Network Pharmacology of GCMs

#### 3.10.1. Screening Active Ingredients and Target Prediction

The Simplified Molecular Input Line Entry System (SMILES) structures of 16 amino acid components, including Asp, Glu, Hyp, Ser, Gly, His, Arg, Thr, Ala, Pro, Tyr, Val, Ile, Leu, Phe, and Lys, were retrieved from the PubChem database (https://pubchem.ncbi.nlm.nih.gov/). These structures were individually imported into the SwissTargetPrediction database (https://swisstargetprediction.ch/ (accessed on 13 February 2025)), with the species parameter set to “Homo sapiens” to predict the potential targets of these compounds. Predicted targets with a “Probability*” value > 0 were selected, and duplicates were removed to obtain the potential targets of the amino acid components. The GeneCards database (https://www.genecards.org/) was searched using the keywords “nourishing”, “tonic”, and “restorative” to identify potential targets related to these effects. Targets with Relevance score > 1 were selected and designated as the restorative active targets. The targets of the components and the restorative targets were imported into Venny 2.1.0 (https://bioinfogp.cnb.csic.es/tools/venny/index.html (accessed on 13 February 2025)) to obtain the overlapping targets as the potential targets of GCMs associated with their restorative effect. Using Cytoscape 3.7.1 software, the overlapping targets and amino acid components were used to construct a “ingredient–target–activity” network.

#### 3.10.2. Protein–Protein Interaction Network

The identified overlapping targets were imported into the STRING database (https://string-db.org) to construct a protein-protein interaction (PPI) network. The species was restricted to “Homo sapiens”, and the “minimum required interaction score” was set to 0.4. The data were imported into Cytoscape 3.7.1 software for visualization and network topology analysis.

#### 3.10.3. GO Functional Enrichment and KEGG Pathway Enrichment Analyses

The DAVID database (https://davidbioinformatics.nih.gov/) was used with the species parameter set to “Homo sapiens”. The targets of GCMs associated with restorative activity were imported, and GO and KEGG enrichment analyses were performed. A *p*-value < 0.05 was considered statistically significant for all analyses. The results were then sorted by *p*-value, and the top 10 terms were selected for visualization.

### 3.11. Data Analysis

The data were presented as mean ± standard deviation (SD) and analyzed using one-way analysis of variance (ANOVA) with SPSS 19.0 (IBM, Armonk, NY, USA). Differences were considered to be statistically significant if *p* < 0.05. Graphs were generated using Origin 9.1 (OriginLab Corp., Northampton, MA, USA). All URLs used in this study were accessed on 13 February 2025. The relative standard deviation (RSD) value reflects the precision of the analytical results in the assay.

## 4. Conclusions

This study systematically investigated the chemical composition, analytical methodology, quality evaluation, and potential pharmacological mechanisms of gelatinous Chinese medicines (GCMs). Using ^1^H NMR spectroscopy, 17 components (16 amino acids and taurine) were identified in acid-hydrolyzed CCA, and comparative profiling of different GCMs revealed only minor compositional differences. With the established PITC pre-column derivatization–HPLC method, most amino acids exhibited good stability under acid hydrolysis, with the exception of sulfur-containing amino acids (cystine and methionine) and tryptophan. An optimized quantitative method for amino acids in GCMs was successfully developed, and when combined with chemometric analysis, it effectively distinguished three types of skin-derived glue and four types of horn-, bone-, or shell-derived glue, providing a foundation for the identification of GCM adulteration. Furthermore, network pharmacology predictions suggested that the tonic activity of GCM amino acids may be associated with 20 core targets (e.g., GRM5, GABRA1, and GRIA2) and key pathways such as neuroactive ligand–receptor interaction and GABAergic synapse. Collectively, this work offers a scientific basis for the quality control of GCMs and contributes to the modern interpretation of their traditional tonic effects.

## Data Availability

Data are contained within the article and Supplementary Materials.

## Supplementary Materials

The following supporting information can be downloaded at https://www.mdpi.com/article/10.3390/molecules31213812/s1, Table S1: The information of different batches of GCMs and EG samples; Table S2: Attribution of ^1^H-NMR signals of chemical components in CCA (600 MHz, D_2_O); Table S3-1: Optimized results of acid hydrolysis temperature of CCA (*n* = 3); Table S3-2: Optimized results of acid hydrolysis time of CCA (*n* = 3); Table S3-3: Optimized results of derivatization reagent phenyl isothiocyanate solution concentration (*n* = 3); Table S3-4: Optimized results of derivatization temperature (*n* = 3); Table S3-5: Optimized results of derivatization time (*n* = 3); Table S4: Normalization results of the peak area of each amino acid under different preparation conditions of the sample solutions (*n* = 3); Table S5: Methodological verification of simultaneous quantification of 16 amino acids in different GCMs; Table S6: Determination results of water content of 65 batches of GCMs (*n* = 3, $\overset{-}{x}\pm \mathrm{SD}$); Table S7: Determination results of amino acid content of 65 batches of GCMs and EG (calculated after deducting dry weight of water content) (*n* = 3, $\overset{-}{x}\pm \mathrm{SD}$); Table S8: The content range of different types of amino acids in GCMs and EG (calculated after deducting dry weight of water content); Table S9: Targets of amino acid; Table S10: Intersection target of “amino acid components-tonic activity”; Table S11: Topological properties of key components; Figure S1: ^1^H NMR spectrum of the acid-hydrolyzed CCA test solution. (A) Full spectrum. Magnified regions: (B) *δ* 0.8–2.8 ppm, (C) *δ* 2.8–4.9 ppm, and (D) *δ* 6.6–8.8 ppm; Figure S2: Chemical structures of amino acids; Figure S3: ^1^H NMR spectrum of the acid-hydrolyzed GCMs test solution. (A) Full spectrum. (B–D) Magnified regions of the spectrum: (B) *δ* 0.8–2.8 ppm, (C) *δ* 2.8–4.9 ppm, and (D) *δ* 6.6–8.8 ppm; Figure S4: Stability of Nor (IS) under hydrochloric acid hydrolysis at different temperatures: (A) 110 °C, (B) 130 °C, and (C) 150 °C (*n* = 3); Figure S5: Optimization of hydrolysis and derivatization conditions for CCA amino acids: effects of (A) hydrolysis temperature, (B) hydrolysis time, (C) PITC concentration, (D) derivatization temperature, and (E) derivatization time on total peak area (*n* = 3); Figure S6: Permutation test results for the OPLS-DA models of different GCM groups: (A) dermal glues, (B) horn and shell glues, (C) CCA, (D) CCB, (E) CCS, (F) CCC, (G) CCPT, (H) CCT, and (I) COC.; Figure S7: Protein-protein interaction diagram of intersection targets.

## Abbreviations

The following abbreviations are used in this manuscript:

GCMs	Gelatinous Chinese medicines
CCA	Colla Corii Asini
CCB	Colla Corii Bovis
CCC	Colla Cornus Cervi
CCPT	Colla Carapacis et Plastri Testudinis
CCS	Colla Corii Suilli
CCT	Colla Carapacis Trionychis
COC	Colla Ossis Cervi
EG	Edible Gelatin
EAA	Essential amino acid
NEAA	Non-essential amino acid
HPLC-PDA	High-performance liquid chromatography with photo-diode array detector
SD	Standard deviation
LOD	Limit of detection
LOQ	Limit of quantitation
S/N	Signal-to-noise ratio
TCM	Traditional Chinese medicine
ChP	Chinese pharmacopoeia
RSD	Relative standard deviation
Gly	Glycine
Pro	L-Proline
Hyp	L-Hydroxyproline
Ala	L-Alanine
Glu	L-Glutamate
Arg	L-Arginine
Asp	L-Aspartic acid
Lys	L-Lysine
Leu	L-Leucine
Ser	L-Serine
Val	L-Valine
Phe	L-Phenylalanine
Ile	L-Isoleucine
Thr	L-Threonine
Tyr	L-Tyrosine
His	L-Hisidine
Met	L-Methionine
Tau	Taurine
Trp	L-Tryptophan
Nor	L-Norleucine
