# Supplementary material for: Integrated NMR, HPLC-Chemometrics, and Network Pharmacology Strategy for Quality Assessment and Mechanism Elucidation of Gelatinous Chinese Medicines
Source: Molecules. 2026 Oct 22;31(21):3812. doi: 10.3390/molecules31213812 (PMC13765498; doi:10.3390/molecules31213812)
Supplement: Supplementary file 1 [file molecules-31-03812-s001.zip › molecules-4601581-supplementary.pdf]

Supplementary Materials

Integrated NMR, HPLC-Chemometrics, and Network Pharmacology Strategy for Quality Assessment and Mechanism Elucidation of Gelatinous Chinese Medicines

Lixin Zhou [1,†], Jianxiao Zheng [1,†], Ying Li [1], Kejing Niu [1], Fuxin Sun [1], Xin Chai [1,2], Yuefei Wang [1,2,*], Huijuan Yu [1,2,*]

[1] State Key Laboratory of Chinese Medicine Modernization, Tianjin University of Traditional Chinese Medicine, Tianjin 301617, China; zhoulixin2623@163.com (L.Z.); z13012621716@163.com (J.Z.); 18303616520@163.com (Y.L.); 18317357226@163.com (K.N.); fuxinsun0801@163.com (F.S.)

[2] Haihe Laboratory of Modern Chinese Medicine, Tianjin 301617, China; chaix0622@tjutcm.edu.cn (X.C.); huijuanyu@tjutcm.edu.cn (H.Y.); wangyf0622@tjutcm.edu.cn (Y.W.)

* Corresponding authors: huijuanyu@tjutcm.edu.cn (H.Y.); wangyf0622@tjutcm.edu.cn (Y.W.); Tel.: +86-22-5959-6366 (H.Y. & Y.W.)

† These authors contributed equally to this work.

Table S1. The information of different batches of GCMs and EG samples

Name	Sample no.	Place of origin	Batch
Colla Corii Asini	CCA1	Dong-E-E-Jiao Co., Ltd.	2309004
	CCA2	Dong-E-E-Jiao Co., Ltd.	2303014
	CCA3	Dong-E-E-Jiao Co., Ltd.	2403016
	CCA4	Dong-E-E-Jiao Co., Ltd.	2405010
	CCA5	Shandong Fupai Ejiao Co., Ltd.	2209042
	CCA6	Shandong Fupai Ejiao Co., Ltd.	2105011
	CCA7	Shandong Fupai Ejiao Co., Ltd.	2109012
	CCA8	Shandong Fupai Ejiao Co., Ltd.	2303017
	CCA9	Shandong Fupai Ejiao Co., Ltd.	2312026
	CCA10	Beijing Tongrentang (Tangshan) Nutrition and Health Products Co., Ltd.	202111113
	CCA11	Beijing Tongrentang (Tangshan) Nutrition and Health Products Co., Ltd.	202311072
	CCA12	Beijing Tongrentang (Tangshan) Nutrition and Health Products Co., Ltd..	202311075
	CCA13	Beijing Tongrentang (Tangshan) Nutrition and Health Products Co., Ltd..	202311080
	CCA14	Shandong Yongletang Ejiao Group Wujie Pharmaceutical Co., Ltd.	01230709
	CCA15	Shandong Yongletang Ejiao Group Wujie Pharmaceutical Co., Ltd.	01231102
	CCA16	Shandong Yongletang Ejiao Group Wujie Pharmaceutical Co., Ltd.	01231103
	CCA17	Jilin Jinuo Pharmaceutical Co., Ltd.	20230701
	CCA18	Jilin Jinuo Pharmaceutical Co. Ltd.	20240501
	CCA19	Hebei Dongru Ejiao Pharmaceutical Co., Ltd.	22050110
	CCA20	Shandong Xijunkang Pharmaceutical Group Xiangtan Co., Ltd.	20211005
	CCA21	Hunan Ai Jingtang Pharmaceutical Co., Ltd.	200801
	CCA22	Shandong Donge Guojiao Tang Ejiao Pharmaceutical Co., Ltd.	221121
	CCA23	Hebei Junlin Pharmaceutical Co. Ltd.	23060717
Colla Corii Bovis	CCB1	Dong-E-E-Jiao Co., Ltd.	2403001
	CCB2	Dong-E-E-Jiao Co., Ltd.	2409001
	CCB3	Henan Sifang Pharmaceutical Group Co., Ltd.	230402
	CCB4	Henan Sifang Pharmaceutical Group Co., Ltd.	240217
	CCB5	Henan Sifang Pharmaceutical Group Co., Ltd.	240301
	CCB6	Henan Fu Ren Tang Pharmaceutical Co., Ltd.	20200503
	CCB7	Henan Fu Ren Tang Pharmaceutical Co., Ltd.	20240309
	CCB8	Baoding Saihang Ejiao Co., Ltd.	0008240902
Colla Cornus Cervi	CCC1	Dong-E-E-Jiao Co., Ltd.	2309002
	CCC2	Dong-E-E-Jiao Co., Ltd.	2309003
	CCC3	Dong-E-E-Jiao Co., Ltd.	2310003
	CCC4	Hubei Kangyuan Pharmaceutical Co., Ltd.	2404211041
	CCC5	Hubei Kangyuan Pharmaceutical Co., Ltd.	2405291036
	CCC6	Hubei Kangyuan Pharmaceutical Co., Ltd.	2405291044
	CCC7	Hunan Ai Jingtang Pharmaceutical Co., Ltd.	220303

	CCC8	Hunan Ai Jingtang Pharmaceutical Co., Ltd.	231205
	CCC9	Hunan Ai Jingtang Pharmaceutical Co., Ltd.	231209
Colla Carapacis et Plastri Testudinis	CCPT1	Dong-E-E-Jiao Co., Ltd.	2312002
	CCPT2	Hunan Ai Jingtang Pharmaceutical Co., Ltd.	200803
	CCPT3	Hunan Ai Jingtang Pharmaceutical Co., Ltd.	221010
	CCPT4	Hunan Ai Jingtang Pharmaceutical Co., Ltd.	221011
	CCPT5	Hubei Kangyuan Pharmaceutical Co., Ltd.	2401051025
	CCPT6	Hubei Kangyuan Pharmaceutical Co., Ltd.	2401051029
	CCPT7	Hubei Kangyuan Pharmaceutical Co., Ltd.	2401051062
	CCPT8	Henan Laojuntang Pharmaceutical Co., Ltd.	230520
	CCPT9	Henan Laojuntang Pharmaceutical Co., Ltd.	230704
Colla Carapacis Trionychis	CCT1	Henan Laojuntang Pharmaceutical Co., Ltd.	220106
	CCT2	Henan Laojuntang Pharmaceutical Co., Ltd.	231209
	CCT3	Henan Laojuntang Pharmaceutical Co., Ltd.	240120
	CCT4	Zhejiang Yifang Pharmaceutical Co., Ltd.	2111002
	CCT5	Zhejiang Yifang Pharmaceutical Co., Ltd.	2208014
Colla Ossis Cervi	COC1	Anhui Bozhou traditional Chinese medicine market	24112701
	COC2	Anhui Bozhou traditional Chinese medicine market	24112702
	COC3	Anhui Bozhou traditional Chinese medicine market	24112703
Colla Corii Suilli	CCS1	Hebei Anguo Chinese Medicinal Materials Market	24061601
	CCS2	Hebei Anguo Chinese Medicinal Materials Market	24061602
	CCS3	Hebei Anguo Chinese Medicinal Materials Market	24061603
	CCS4	Hebei Anguo Chinese Medicinal Materials Market	24061604
edible gelatin	EG1	Shangshui Fuyuan Gelatin Co., Ltd.	240708
	EG2	Wankang Biotechnology Co., Ltd.	240812
	EG3	Shangshui Fuyuan Gelatin Co., Ltd.	240904
	EG4	Shangshui Fuyuan Gelatin Co., Ltd.	241022

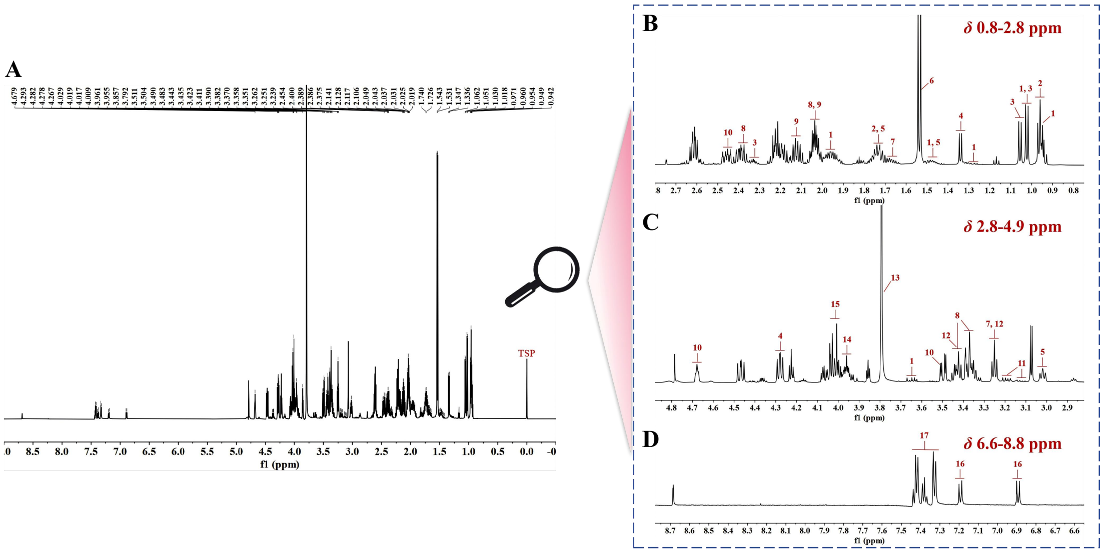

Figure S1. ^{1}H NMR spectrum of the acid-hydrolyzed CCA test solution. (A) Full spectrum. Magnified regions: (B) δ 0.8-2.8 ppm, (C) δ 2.8-4.9 ppm, and (D) δ 6.6-8.8 ppm.

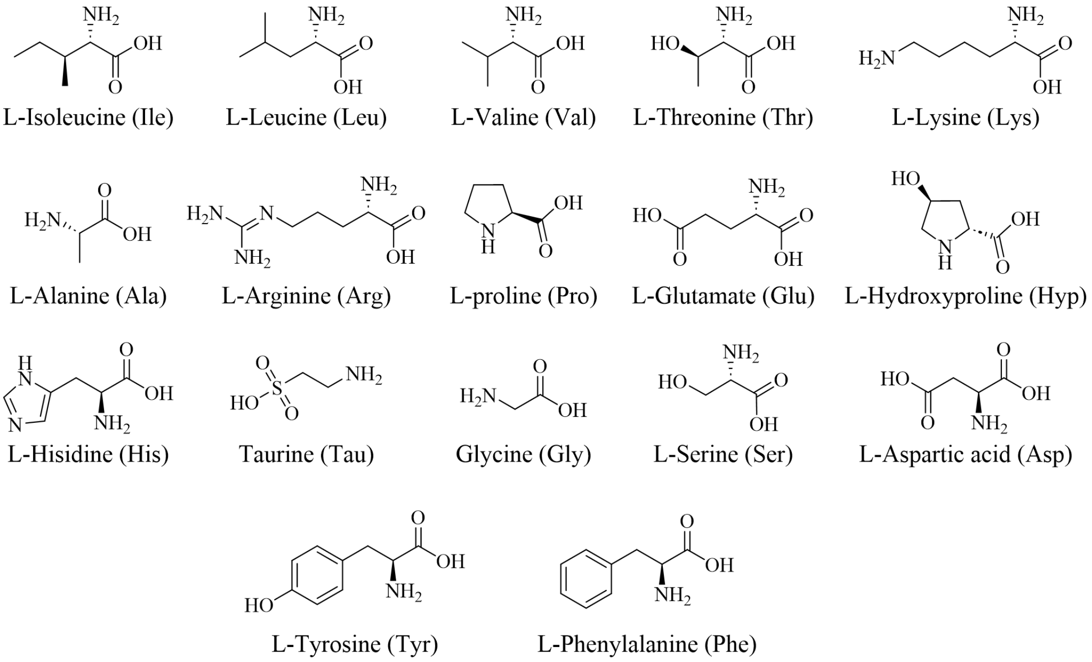

Figure S2. Chemical structures of amino acids.

Table S2. Attribution of ^{1}H-NMR signals of chemical components in CCA (600 MHz, D_2O)

Serial number	name	Signal attribution	Chemical shift in samples (ppm)	Chemical shift in reference standards (ppm)
1	Ile	δ-CH_3	0.94 (t, *J*=7.2 Hz)	0.94 (t, *J*=7.4 Hz)
		γ'-CH_3	1.02 (d, *J*=7.0 Hz)	1.01 (d, *J*=7.0 Hz)
		γ-CH_2	1.26 (m), 1.47 (m)	1.27 (m), 1.48 (m)
		β-CH	1.96 (m)	1.98 (m)
		α-CH	3.66 (m)	3.67 (d, *J*=4.0 Hz)
2	Leu	δ, δ'-CH_3	0.96 (t, *J*=6.8 Hz)	0.96 (t, *J*=6.4 Hz)
		β-CH_2	1.72 (m)	1.72 (m)
3	Val	γ'-CH_3	1.02 (d, *J*=7.0 Hz)	0.99 (d, *J*=7.0 Hz)
		γ-CH_3	1.06 (d, *J*=7.0 Hz)	1.05 (d, *J*=7.0 Hz)
		β-CH	2.33 (m)	2.28 (m)
4	Thr	γ-CH_3	1.34 (d, *J*=6.7 Hz)	1.33 (d, *J*=6.6 Hz)
		β-CH	4.28 (m)	4.25 (m)
5	Lys	γ-CH_2	1.48 (m)	1.44 (m)
		δ-CH_3	1.73 (m)	1.71 (m)
		ε-CH_2	3.02 (t, *J*=7.7 Hz)	3.00 (t, *J*=7.5 Hz)
6	Ala	β-CH_3	1.54 (d, *J*=7.3 Hz)	1.48（d, *J*=7.3 Hz)
7	Arg	γ-CH_2	1.67 (m)	1.63 (m)
		δ-CH_2	3.25 (t, *J*=6.9 Hz)	3.21 (t, *J*=6.6 Hz)
8	Pro	γ-CH_2	2.03 (m)	2.05 (m)
		β-CH_2	2.39 (m)	2.35 (m)
		δ-CH_2	3.37 (m), 3.43 (m)	3.34 (m), 3.43(m)
9	Glu	β-CH_2	2.13 (m)	2.14 (m)
		γ-CH_2	2.52 (m)	2.54 (m)
10	Hyp	β-CH_2	2.18 (m), 2.46 (m)	2.17 (m), 2.44 (m)
		δ-CH_2	3.50 (dd, *J*=12.6, 3.8 Hz)	3.49 (dd, *J*=12.6, 3.8 Hz)
		γ-CH	4.68 (t, *J*=4.2 Hz)	4.67 (t, *J*=4.2 Hz)
		α-CH	4.37 (m)	4.35 (m)
11	His	β-CH_2	3.12 (dd, *J*=14.9, 7.8 Hz)	3.12 (dd, *J*=15.5, 4.7 Hz)
			3.19 (dd, *J*=14.6, 7.8 Hz)	3.23 (dd, *J*=15.5, 8.0 Hz)
12	Tau	α-CH_2	3.25 (t, *J*=6.9 Hz)	3.26 (t, *J*=6.6 Hz)
		β-CH_2	3.43 (m)	3.43 (t, *J*=6.6 Hz)
13	Gly	α-CH_2	3.79 (s)	3.56 (s)
14	Ser	β-CH_2	3.96 (m)	3.97 (m)
15	Asp	α-CH	4.02 (m)	4.03 (m)
		β-CH_2	3.04 (dd)	3.00 (dd)
16	Tyr	Ar-H	6.89 (d, *J*=8.5 Hz), 7.19 (d, *J*=8.5 Hz)	6.90 (d, *J*=8.5 Hz), 7.20 (d, *J*=8.5 Hz)
		α-CH	3.94 (dd)	3.94 (dd)
		β-CH_2	3.04 (dd), 3.19 (dd)	3.06 (dd), 3.21 (dd)
17	Phe	Ar-H	7.33 (m), 7.38 (m), 7.43 (m)	7.34 (m), 7.38 (m), 7.43 (m)
		α-CH	4.02 (dd)	4.00 (dd)
		β-CH_2	3.12 (dd)	3.13 (dd)

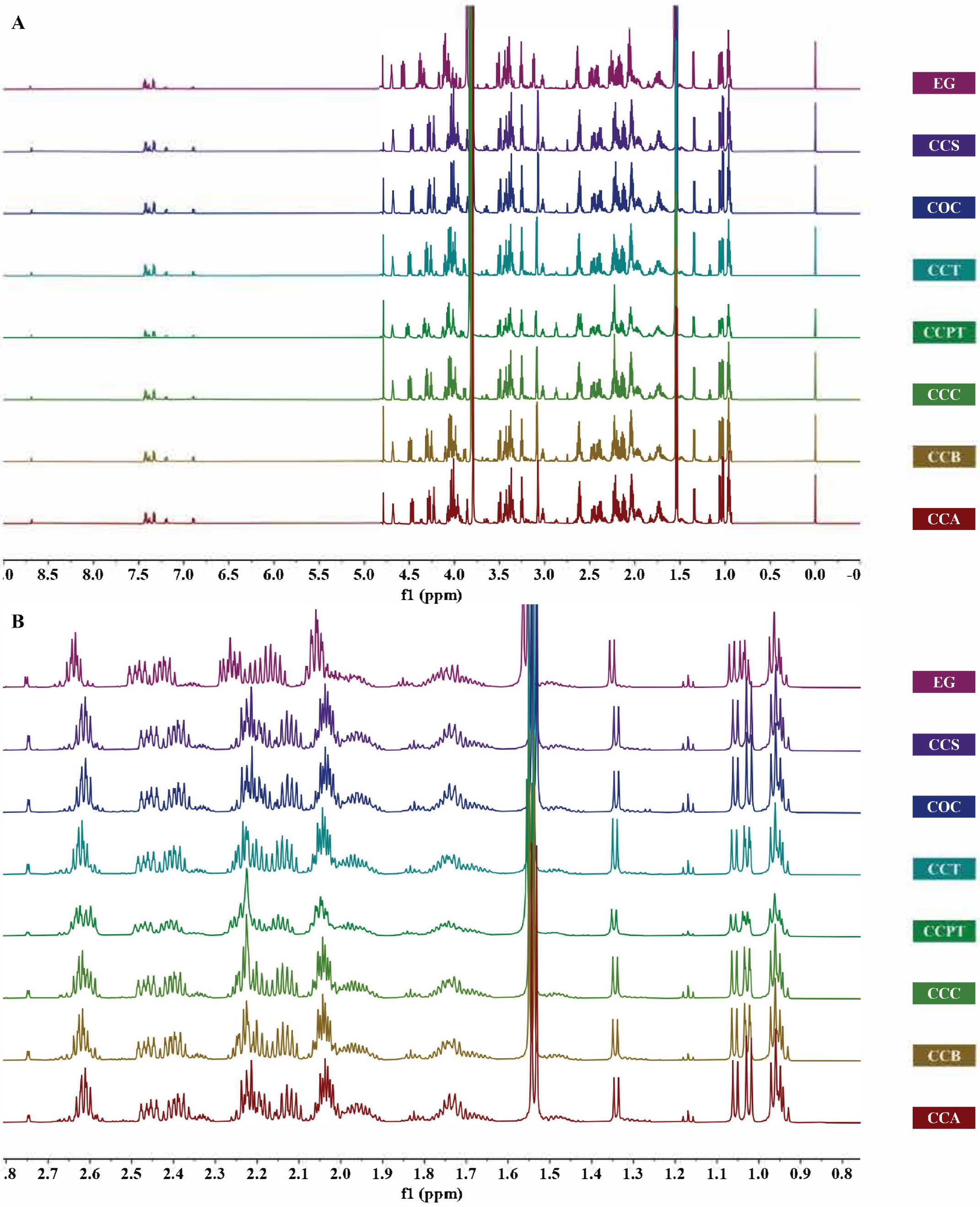
A
EG
CCS
COC
CCT
CCPT
CCC
CCB
CCA
f1 (ppm)
B
EG
CCS
COC
CCT
CCPT
CCC
CCB
CCA
f1 (ppm)

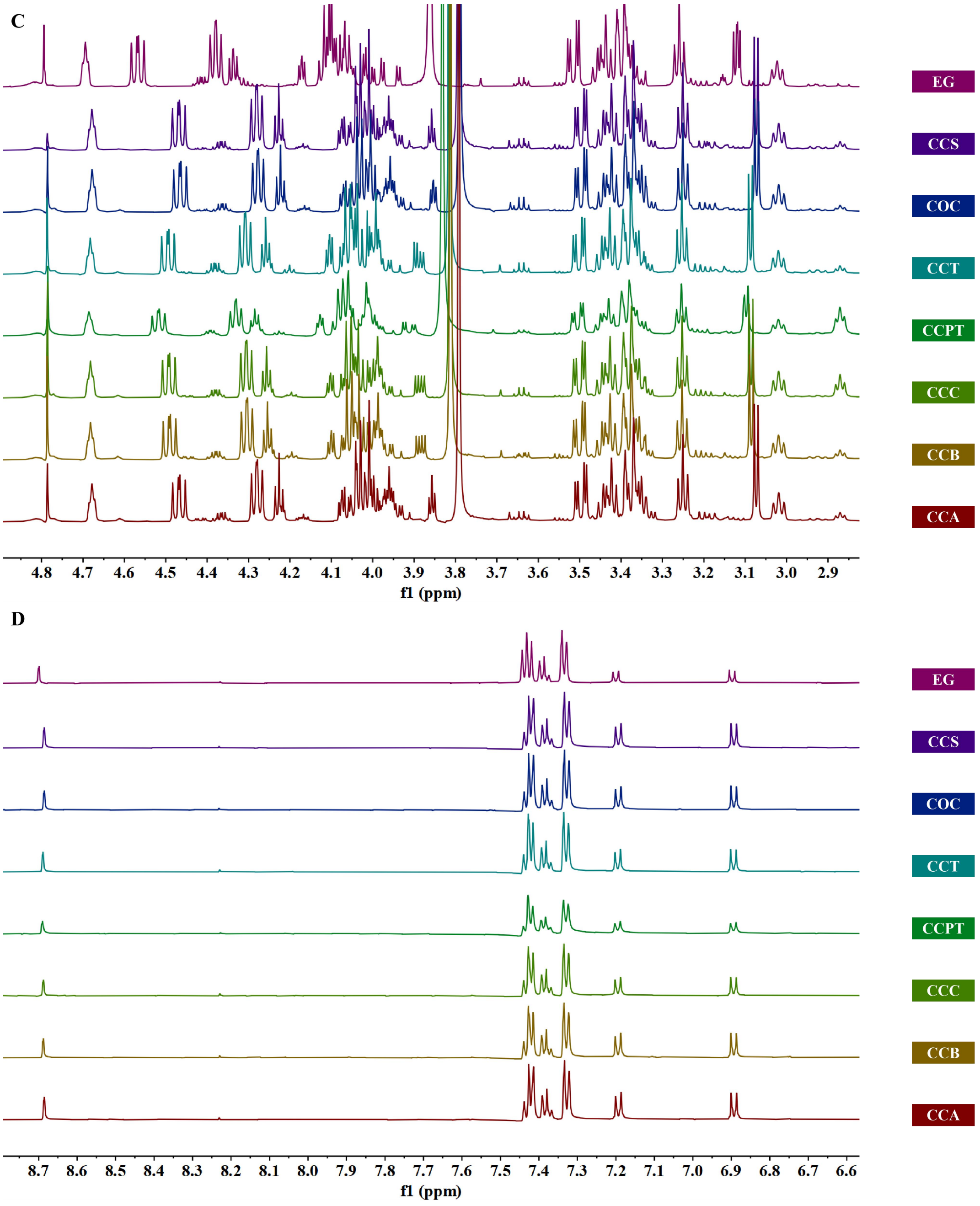

Figure S3. ^1H NMR spectrum of the acid-hydrolyzed GCMs test solution. (A) Full spectrum. (B-D) Magnified regions of the spectrum: (B) δ 0.8-2.8 ppm, (C) δ 2.8-4.9 ppm, and (D) δ 6.6-8.8 ppm.

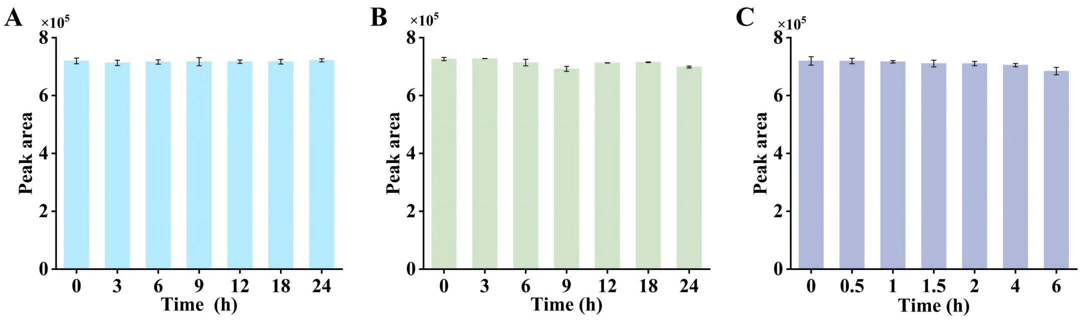

Figure S4. Stability of Nor (IS) under hydrochloric acid hydrolysis at different temperatures: (A) 110 °C, (B) 130 °C, and (C) 150 °C (*n*=3).

Table S3-1. Optimized results of acid hydrolysis temperature of CCA (n=3)

**Chemical compound**	**Peak area per unit mass (A/mg)**		
	**110 ℃**	**130 ℃**	**150 ℃**
Asp	8865	12717	13514
Glu	16025	23276	24273
Hyp	34366	57027	58732
Ser	15346	18621	16661
Gly	155329	192679	194682
His	1259	1993	2342
Arg	11852	20613	22771
Thr	3502	5552	5587
Ala	42691	55572	56335
Pro	44158	74326	78459
Tyr	1513	2679	3064
Val	2516	7785	13981
Ile	1824	4793	8025
Leu	9295	15415	17280
Phe	4031	6539	7864
Lys	13736	22755	26198
Total peak area	366308	522343	549768

Table S3-2. Optimized results of acid hydrolysis time of CCA (n=3)

**Chemical compound**	**Peak area per unit mass (A/mg)**				
	**1 h**	**2 h**	**3 h**	**4 h**	**5 h**
Asp	3952	12109	14020	14634	14726
Glu	6988	23139	25316	26078	26023
Hyp	8310	53540	60727	60407	55636
Ser	6264	18830	17430	16729	13793
Gly	63780	190627	203026	209027	205962
His	414	1634	2192	2136	2079
Arg	2424	19588	23570	23843	23652
Thr	3723	4462	5218	4834	4243
Ala	16907	54170	56991	58122	62057
Pro	11646	70095	81569	81407	80102
Tyr	414	2175	2934	3162	2826
Val	697	6261	14188	15135	15661
Ile	407	3175	7463	7714	7654
Leu	2982	13516	16906	17226	17148
Phe	734	6031	6955	6571	6445
Lys	4244	20663	26606	26781	28255
Total peak area	133887	500015	565112	573805	566264

Table S3-3. Optimized results of derivatization reagent phenyl isothiocyanate solution concentration (n=3)

Chemical compound	Peak area per unit mass (A/mg)				
	**0.1 mol/L**	**0.2 mol/L**	**0.3 mol/L**	**0.4 mol/L**	**0.5 mol/L**
Asp	13675	25882	28202	28219	28674
Glu	24736	43118	45401	45238	44767
Hyp	61869	63260	63584	63677	63745
Ser	16887	24563	24793	24492	24116
Gly	204021	215151	215275	215613	217152
His	2129	2930	2776	2857	2781
Arg	23458	32031	32276	32108	31983
Thr	4380	7079	6880	6938	6469
Ala	56026	71644	71319	70960	70239
Pro	81646	82793	82359	82299	82363
Tyr	3050	3073	3040	2899	2846
Val	14835	16953	16833	16571	16356
Ile	7293	7923	7802	7552	7177
Leu	16819	18331	18266	18029	17467
Phe	6544	6947	6822	6549	6360
Lys	26259	28746	28708	27386	26771
Total peak area	563627	650421	654336	651386	652005

Table S3-4. Optimized results of derivatization temperature (n=3)

**Chemical compound**	**Peak area per unit mass (A/mg)**			
	**25℃**	**35℃**	**45℃**	**55℃**
Asp	25122	29250	29027	27876
Glu	42779	47609	47302	46048
Hyp	63471	63577	63584	64394
Ser	25158	26170	26107	25865
Gly	215315	215891	216126	217967
His	3339	3478	3479	3429
Arg	32220	33017	32669	31921
Thr	9727	10557	10684	10709
Ala	72698	73916	73518	72590
Pro	84754	84769	84898	86001
Tyr	3318	3345	3279	3237
Val	16999	17230	16911	16208
Ile	9518	9586	9440	9104
Leu	19980	20109	19931	19434
Phe	9339	9413	9371	9254
Lys	27573	27688	27970	27692
Total peak area	661309	675605	674292	671729

Table S3-5. Optimized results of derivatization time (n=3)

**Chemical compound**	**Peak area per unit mass (A/mg)**				
	**20 min**	**40 min**	**60 min**	**80 min**	**100 min**
Asp	25877	28308	29024	28660	28449
Glu	44048	46638	47401	46776	46452
Hyp	63211	63593	64298	63831	63726
Ser	25481	25855	26125	25811	25681
Gly	213847	214876	217413	216118	215471
His	3389	3414	3445	3438	3398
Arg	32364	32647	32831	32413	32364
Thr	10168	10313	10394	10223	10119
Ala	72975	73214	73754	72972	72511
Pro	84050	84522	85702	85422	85439
Tyr	3339	3323	3354	3317	3290
Val	17152	17019	17226	16918	16701
Ile	9606	9571	9605	9359	9182
Leu	20019	20041	20166	19961	19835
Phe	9375	9424	9447	9332	9265
Lys	27148	27617	28420	28313	28043
Total peak area	662048	670376	678605	672864	669927

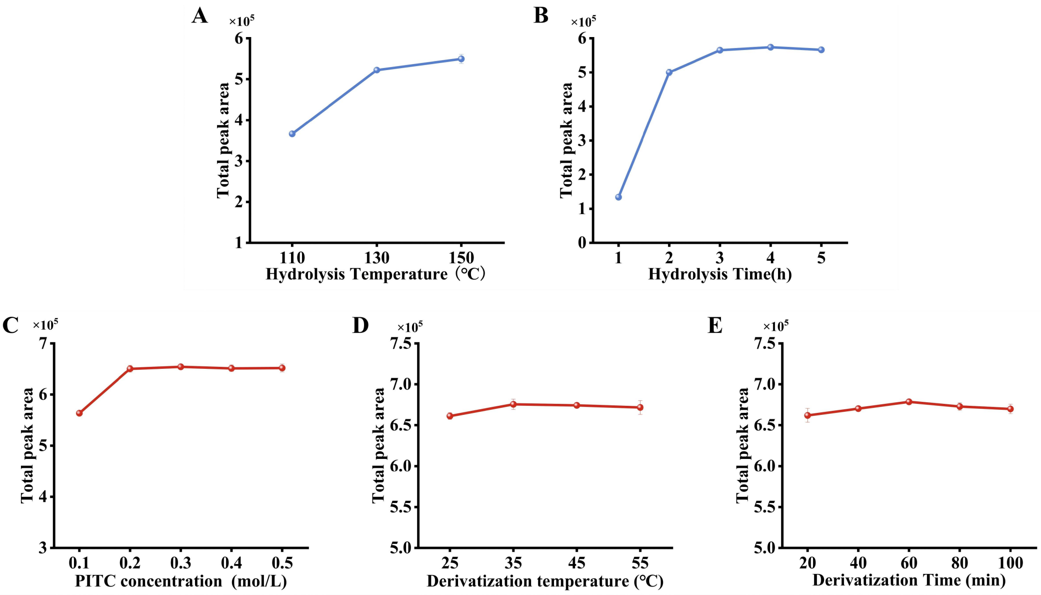

Figure S5. Optimization of hydrolysis and derivatization conditions for CCA amino acids: effects of (A) hydrolysis temperature, (B) hydrolysis time, (C) PITC concentration, (D) derivatization temperature, and (E) derivatization time on total peak area (*n* = 3).

Table S4. Normalization results of the peak area of each amino acid under different preparation conditions of the sample solutions (*n*=3)

	**Element $E_{m\text{-}j\text{-}k}$**	**Asp**	**Glu**	**Hyp**	**Ser**	**Gly**	**His**	**Arg**	**Thr**	**Ala**	**Pro**	**Tyr**	**Val**	**Ile**	**Leu**	**Phe**	**Lys**	***S***
Temperature of hydrolysis	110 ℃	0.6560	0.6602	0.5851	0.8241	0.7979	0.5377	0.5205	0.6268	0.7578	0.5628	0.4940	0.1800	0.2273	0.5379	0.5126	0.5243	0.89
	130 ℃	0.9411	0.9589	0.9710	1.0000	0.9897	0.8509	0.9053	0.9938	0.9865	0.9473	0.8743	0.5568	0.5973	0.8921	0.8315	0.8686	2.17
	150 ℃	1.0000	1.0000	1.0000	0.8948	1.0000	1.0000	1.0000	1.0000	1.0000	1.0000	1.0000	1.0000	1.0000	1.0000	1.0000	1.0000	2.64
Time of hydrolysis	1 h	0.2684	0.2680	0.1368	0.3327	0.3051	0.1891	0.1017	0.7135	0.2724	0.1428	0.1309	0.0445	0.0528	0.1731	0.1056	0.1502	0.10
	2 h	0.8223	0.8873	0.8817	1.0000	0.9120	0.7454	0.8215	0.8551	0.8729	0.8593	0.6880	0.3998	0.4116	0.7846	0.8671	0.7313	1.73
	3 h	0.9520	0.9708	1.0000	0.9257	0.9713	1.0000	0.9885	1.0000	0.9184	1.0000	0.9280	0.9059	0.9675	0.9815	1.0000	0.9416	2.51
	4 h	0.9937	1.0000	0.9947	0.8884	1.0000	0.9744	1.0000	0.9265	0.9366	0.9980	1.0000	0.9664	1.0000	1.0000	0.9448	0.9478	2.53
	5 h	1.0000	0.9979	0.9162	0.7325	0.9853	0.9483	0.9920	0.8132	1.0000	0.9820	0.8939	1.0000	0.9923	0.9955	0.9267	1.0000	2.37
Concentration of PITC	0.1 M	0.4769	0.5448	0.9706	0.6811	0.9395	0.7268	0.7268	0.6188	0.7820	0.9862	0.9926	0.8751	0.9205	0.9175	0.9419	0.9135	1.74
	0.2 M	0.9026	0.9497	0.9924	0.9907	0.9908	1.0000	0.9924	1.0000	1.0000	1.0000	1.0000	1.0000	1.0000	1.0000	1.0000	1.0000	2.61
	0.3 M	0.9835	1.0000	0.9975	1.0000	0.9914	0.9475	1.0000	0.9719	0.9955	0.9948	0.9894	0.9929	0.9847	0.9964	0.9820	0.9987	2.62
	0.4 M	0.9841	0.9964	0.9989	0.9879	0.9929	0.9751	0.9948	0.9801	0.9905	0.9940	0.9435	0.9775	0.9531	0.9835	0.9427	0.9527	2.56
	0.5 M	1.0000	0.9860	1.0000	0.9727	1.0000	0.9493	0.9909	0.9138	0.9804	0.9948	0.9263	0.9648	0.9059	0.9529	0.9155	0.9313	2.48
Temperature of derivatization	25 ℃	0.8589	0.8986	0.9857	0.9613	0.9878	0.9597	0.9759	0.9083	0.9835	0.9855	0.9919	0.9866	0.9928	0.9936	0.9921	0.9858	2.48
	35 ℃	1.0000	1.0000	0.9873	1.0000	0.9905	0.9997	1.0000	0.9857	1.0000	0.9857	1.0000	1.0000	1.0000	1.0000	1.0000	0.9899	2.66
	45 ℃	0.9924	0.9935	0.9874	0.9976	0.9915	1.0000	0.9894	0.9976	0.9946	0.9872	0.9802	0.9815	0.9848	0.9911	0.9954	1.0000	2.63
	55 ℃	0.9530	0.9672	1.0000	0.9883	1.0000	0.9857	0.9668	1.0000	0.9821	1.0000	0.9678	0.9407	0.9497	0.9664	0.9830	0.9901	2.57
Time of derivatization	20 min	0.8916	0.9293	0.9831	0.9754	0.9836	0.9838	0.9858	0.9782	0.9894	0.9807	0.9957	0.9957	1.0000	0.9927	0.9924	0.9552	2.54
	40 min	0.9753	0.9839	0.9890	0.9897	0.9883	0.9911	0.9944	0.9923	0.9927	0.9862	0.9907	0.9880	0.9964	0.9938	0.9976	0.9717	2.62
	60 min	1.0000	1.0000	1.0000	1.0000	1.0000	1.0000	1.0000	1.0000	1.0000	1.0000	1.0000	1.0000	0.9999	1.0000	1.0000	1.0000	2.68
	80 min	0.9875	0.9868	0.9927	0.9880	0.9940	0.9980	0.9873	0.9836	0.9894	0.9967	0.9892	0.9821	0.9744	0.9898	0.9877	0.9962	2.62
	100 min	0.9802	0.9800	0.9911	0.9830	0.9911	0.9864	0.9858	0.9735	0.9831	0.9969	0.9810	0.9695	0.9559	0.9836	0.9807	0.9867	2.59

Table S5. Methodological verification of simultaneous quantification of 16 amino acids in different GCMs

amino acid	regression equation	r^2	Liner range (μg/mL)	LODs (μg/mL)	LoQs (μg/mL)	Instrumental repeatability (RSD, *n*=6, %)	Instrumental inter-day precisio instrumental inter-day precisio (RSD, *n*=6, %)	Stability (RSD, *n*=8, %)	Independent sample-preparation repeatability (RSD, *n*=6, %)	Recovery	
										spike recovery (*n*=6, %)	RSD (%)
Asp	$y = 61790x - 22897$	0.999	0.7184 – 45.98	0.0266	0.0798	0.19	2.09	0.75	1.16	91.32	2.98
Glu	$y = 54930x - 28153$	0.999	1.315 – 84.18	0.0487	0.1461	0.20	1.86	0.68	0.69	95.67	2.80
Hyp	$y = 63741x + 7874.3$	0.999	1.430 – 91.52	0.0530	0.1589	0.15	0.13	0.21	0.91	107.3	1.42
Ser	$y = 83442x - 12247$	0.999	0.4912 – 31.44	0.0182	0.0546	0.30	0.84	0.56	2.05	99.99	2.52
Gly	$y = 119690x - 8621.7$	0.999	2.926 – 187.3	0.1084	0.3251	0.12	0.23	0.14	0.58	90.74	4.21%
His	$y = 57647x - 1809.2$	0.999	0.0977 – 6.225	0.0109	0.0326	0.35	2.17	1.03	1.64	101.4	2.13
Arg	$y = 42185x - 14996$	0.999	1.180 – 75.55	0.0437	0.1312	0.33	0.99	0.64	0.78	104.6	2.55
Thr	$y = 73446x - 10921$	0.999	0.2309 – 14.78	0.0086	0.0257	0.20	1.85	0.56	0.39	104.0	2.04
Ala	$y = 98713x - 22588$	0.999	1.180 – 75.55	0.1312	0.3935	0.14	1.57	0.56	1.09	104.8	2.86
Pro	$y = 76547x + 8298.4$	0.999	1.638 – 104.8	0.0607	0.1820	0.10	0.47	0.22	0.60	102.7	2.83
Tyr	$y = 50225x - 1326.3$	0.999	0.1093 – 6.994	0.0121	0.0364	0.41	0.89	0.88	1.84	99.86	2.61
Val	$y = 81906x - 6348.7$	0.999	0.3332 – 21.32	0.0123	0.0370	0.38	2.71	1.09	2.60	95.82	3.84
Ile	$y = 74642x + 4198.2$	0.999	0.2285 – 14.62	0.0085	0.0254	0.29	2.51	0.90	2.79	101.8	2.34
Leu	$y = 75851x - 7840.1$	0.999	0.4266 – 27.30	0.0158	0.0474	0.20	1.70	0.69	1.41	96.56	2.47
Phe	$y = 56447x - 2234.2$	0.999	0.2652 – 16.98	0.0295	0.0884	0.29	2.11	0.84	1.12	102.7	2.59
Lys	$y = 77368x - 25023$	0.999	0.5984 – 38.30	0.0222	0.0665	0.18	1.64	0.65	2.92	109.3	1.22

Table S6. Determination results of water content of 65 batches of GCMs (n=3, $\bar{x} \pm SD$)

**batch**	**Water content (%)**	**batch**	**Water content (%)**
CCA1	7.85 ± 0.17	CCC3	8.77 ± 0.09
CCA2	11.86 ± 0.09	CCC4	11.20 ± 0.90
CCA3	11.35 ± 0.23	CCC5	11.28 ± 0.82
CCA4	12.24 ± 0.20	CCC6	10.35 ± 0.21
CCA5	11.28 ± 0.35	CCC7	10.68 ± 0.74
CCA6	10.68 ± 1.08	CCC8	12.02 ± 0.52
CCA7	9.70 ± 0.24	CCC9	11.18 ± 0.60
CCA8	9.09 ± 0.35	CCPT1	3.38 ± 0.34
CCA9	10.80 ± 0.64	CCPT2	12.09 ± 0.58
CCA10	8.76 ± 0.58	CCPT3	12.05 ± 0.58
CCA11	9.98 ± 0.55	CCPT4	12.47 ± 0.47
CCA12	9.51 ± 0.16	CCPT5	12.17 ± 0.61
CCA13	9.72 ± 0.71	CCPT6	11.52 ± 0.35
CCA14	9.89 ± 0.13	CCPT7	11.67 ± 0.37
CCA15	10.97 ± 0.15	CCPT8	10.05 ± 0.30
CCA16	10.84 ± 0.56	CCPT9	12.46 ± 0.65
CCA17	8.86 ± 0.68	CCT1	6.24 ± 0.18
CCA18	10.07 ± 0.92	CCT2	12.01 ± 0.63
CCA19	11.17 ± 0.69	CCT3	12.05 ± 0.57
CCA20	9.63 ± 0.30	CCT4	10.79 ± 0.72
CCA21	11.69 ± 0.44	CCT5	10.17 ± 0.52
CCA22	12.11 ± 0.56	COC1	11.65 ± 0.36
CCA23	8.86 ± 0.68	COC2	11.89 ± 0.51
CCB1	6.82 ± 0.44	COC3	10.64 ± 0.30
CCB2	12.06 ± 0.38	CCS1	7.57 ± 0.60
CCB3	11.25 ± 0.37	CCS2	9.35 ± 0.56
CCB4	11.42 ± 0.34	CCS3	9.10 ± 0.32
CCB5	12.26 ± 0.46	CCS4	8.92 ± 0.50
CCB6	11.94 ± 0.12	EG1	12.78 ± 0.22
CCB7	12.21 ± 0.69	EG2	11.60 ± 0.08
CCB8	10.93 ± 0.63	EG3	11.93 ± 0.42
CCC1	9.73 ± 0.70	EG4	10.91 ± 0.22
CCC2	5.25 ± 0.10	–	–

Table S7. Determination results of amino acid content of 65 batches of GCMs and EG (calculated after deducting dry weight of water content) (*n*=3, $\bar{x}\pm$SD)

Beaches	Content（mg/g）															
	**Asp**	**Glu**	**Hyp**	**Ser**	**Gly**	**His**	**Arg**	**Thr**	**Ala**	**Pro**	**Tyr**	**Val**	**Ile**	**Leu**	**Phe**	**Lys**
CCA1	52.11	95.47	108.24	35.73	195.53	6.484	85.74	16.86	81.67	118.72	7.485	22.47	13.18	28.91	18.34	40.14
	±0.55	±0.60	±0.90	±0.67	±1.03	±0.10	±0.61	±0.06	±0.82	±7.49	±0.13	±0.53	±0.34	±0.37	±0.19	±1.07
CCA2	52.06	95.64	105.93	35.25	193.04	6.661	86.70	17.04	81.40	117.60	7.525	23.29	14.00	29.27	18.46	40.49
	±0.35	±0.64	±1.30	±0.59	±2.24	±0.02	±0.92	±0.21	±0.21	±1.24	±0.07	±0.16	±0.17	±0.20	±0.19	±1.07
CCA3	52.13	95.76	104.00	34.49	190.44	6.650	86.37	16.67	81.71	114.97	7.401	23.99	14.23	28.96	18.43	42.58
	±0.22	±0.22	±0.69	±0.46	±0.23	±0.09	±0.11	±0.05	±0.43	±0.27	±0.05	±0.41	±0.25	±0.12	±0.08	±1.00
CCA4	50.81	93.72	103.33	35.25	186.78	6.574	85.68	16.85	79.26	113.68	7.556	21.91	13.07	28.65	18.47	41.66
	±0.56	±0.98	±0.15	±0.14	±0.46	±0.11	±0.44	±0.11	±0.51	±0.10	±0.14	±0.56	±0.36	±0.22	±0.14	±0.83
CCA5	51.71	95.43	105.21	33.86	191.84	6.297	83.49	16.47	81.92	117.42	7.286	23.51	13.88	28.94	18.16	40.29
	±0.35	±0.90	±1.53	±0.66	±1.70	±0.15	±0.61	±0.29	±0.60	±1.19	±0.12	±0.25	±0.09	±0.26	±0.15	±0.85
CCA6	49.51	91.65	103.47	34.81	186.17	6.401	81.63	16.24	78.10	114.30	7.531	21.61	12.89	28.27	17.99	40.69
	±0.42	±0.62	±0.80	±0.22	±1.37	±0.01	±0.61	±0.12	±0.55	±0.81	±0.05	±0.40	±0.24	±0.19	±0.07	±0.46
CCA7	49.34	90.94	99.19	33.05	181.25	6.431	78.95	16.03	77.24	111.94	7.738	22.69	13.58	28.73	17.80	39.70
	±0.38	±0.94	±2.04	±0.80	±2.37	±0.05	±0.97	±0.29	±0.80	±1.72	±0.15	±0.14	±0.12	±0.34	±0.31	±0.68
CCA8	49.43	93.83	109.46	35.91	193.75	6.042	82.71	16.36	80.82	118.64	7.343	19.70	11.54	28.37	18.15	39.91
	±0.46	±0.94	±1.68	±0.71	±2.45	±0.07	±0.71	±0.17	±0.95	±1.54	±0.07	±0.54	±0.34	±0.21	±0.16	±0.22
CCA9	52.13	95.64	95.52	24.71	194.09	6.319	80.80	14.36	89.62	119.12	6.941	24.85	14.76	29.96	18.36	43.10
	±0.69	±1.11	±1.52	±1.14	±2.37	±0.04	±0.92	±0.28	±2.28	±1.45	±0.06	±0.33	±0.17	±0.40	±0.24	±0.96
CCA10	43.87	80.52	97.17	30.87	172.03	5.461	72.95	14.38	70.53	105.17	6.046	18.43	10.75	23.72	15.52	35.68
	±0.26	±0.44	±0.97	±0.24	±1.04	±0.03	±0.47	±0.10	±0.55	±0.88	±0.04	±0.04	±0.06	±0.15	±0.13	±0.64
CCA11	45.07	81.95	96.17	30.24	173.86	5.813	74.41	14.47	71.98	105.95	6.332	20.01	11.93	24.47	15.76	37.54
	±0.69	±0.90	±0.25	±0.17	±1.46	±0.09	±0.82	±0.06	±1.00	±0.54	±0.07	±0.51	±0.24	±0.29	±0.11	±0.93
CCA12	41.99	77.91	96.17	30.85	169.49	5.116	70.41	13.86	68.86	103.99	5.792	16.37	9.41	22.88	15.21	34.93
	±2.01	±3.53	±4.18	±1.27	±7.08	±0.24	±3.38	±0.63	±3.05	±4.52	±0.34	±1.04	±0.65	±1.10	±0.81	±3.21
CCA13	45.06	81.12	86.52	22.38	173.76	5.768	73.41	12.46	77.61	106.20	6.095	20.73	11.98	24.31	15.38	38.31
	±0.69	±1.22	±2.57	±1.14	±3.23	±0.09	±1.33	±0.36	±1.30	±2.05	±0.21	±0.35	±0.24	±0.48	±0.43	±2.03

CCA14	48.14	91.34	109.27	32.84	191.41	5.395	78.02	16.37	81.52	117.90	6.553	21.12	11.58	27.07	18.03	36.48
	±0.49	±1.16	±1.05	±0.46	±2.13	±0.08	±0.76	±0.15	±0.66	±1.15	±0.11	±0.46	±0.33	±0.28	±0.22	±0.12
CCA15	40.45	79.68	97.56	29.23	168.89	4.281	63.30	13.92	72.31	104.77	5.353	16.73	8.91	23.35	15.67	27.35
	±0.37	±1.10	±1.65	±0.43	±2.71	±0.05	±0.68	±0.18	±1.03	±1.84	±0.03	±0.18	±0.16	±0.26	±0.13	±0.06
CCA16	43.45	81.59	85.25	19.16	172.50	4.661	65.80	11.99	82.16	106.21	5.328	21.45	11.57	24.39	16.06	32.41
	±0.73	±1.04	±2.35	±1.46	±1.45	±0.02	±0.82	±0.36	±1.45	±0.77	±0.15	±0.14	±0.10	±0.29	±0.21	±0.88
CCA17	47.05	87.96	90.88	21.56	180.21	5.077	72.84	13.21	86.29	110.87	5.995	23.54	12.12	26.64	17.27	36.30
	±0.35	±0.74	±2.89	±1.48	±1.63	±0.10	±0.89	±0.49	±1.18	±1.12	±0.08	±0.05	±0.09	±0.19	±0.07	±0.27
CCA18	47.62	88.85	97.20	25.37	184.03	5.077	74.47	14.18	84.71	113.05	6.236	23.55	12.27	27.10	17.76	40.27
	±0.45	±0.69	±1.30	±0.77	±1.99	±0.09	±0.87	±0.18	±1.72	±1.25	±0.22	±0.38	±0.26	±0.47	±0.61	±6.23
CCA19	47.45	87.91	96.71	31.30	184.00	5.937	78.51	14.53	77.81	114.85	6.634	22.21	13.36	26.91	17.04	38.15
	±0.52	±1.03	±1.90	±0.86	±2.18	±0.02	±0.89	±0.25	±0.64	±1.49	±0.06	±0.07	±0.08	±0.25	±0.13	±0.46
CCA20	51.33	94.62	99.68	26.95	194.47	6.185	85.38	14.88	88.16	118.55	6.900	24.51	13.66	28.98	18.15	42.97
	±0.05	±0.16	±1.30	±0.81	±0.45	±0.02	±0.32	±0.21	±0.86	±0.49	±0.11	±0.13	±0.07	±0.05	±0.07	±0.50
CCA21	45.99	88.48	101.41	31.62	186.88	5.481	73.50	14.06	78.03	117.59	6.482	20.89	12.11	26.22	16.88	33.66
	±1.12	±2.10	±1.43	±0.70	±3.29	±0.16	±1.18	±0.30	±1.37	±1.68	±0.19	±0.29	±0.30	±0.58	±0.43	±1.36
CCA22	52.80	97.10	111.28	36.44	197.97	6.605	86.35	17.20	82.61	120.36	7.515	22.49	13.11	29.18	18.60	42.00
	±0.67	±1.16	±1.25	±0.33	±2.16	±0.09	±0.99	±0.18	±0.95	±1.39	±0.17	±0.48	±0.27	±0.39	±0.31	±1.84
CCA23	46.24	86.25	84.66	20.06	174.41	5.982	72.59	13.20	84.37	108.05	6.284	23.60	11.83	26.95	17.52	38.45
	±0.32	±0.55	±1.34	±1.10	±1.15	±0.03	±0.22	±0.30	±1.81	±0.71	±0.07	±0.20	±0.09	±0.14	±0.07	±0.35
CCB1	48.91	87.93	100.28	29.03	184.21	5.905	82.98	14.99	78.07	113.48	6.290	19.76	13.35	25.51	15.43	39.01
	±0.75	±1.02	±0.50	±0.14	±2.07	±0.04	±0.74	±0.08	±1.36	±1.07	±0.13	±0.57	±0.40	±0.35	±0.31	±1.34
CCB2	46.85	86.55	100.94	29.57	182.15	5.551	76.82	14.72	76.70	113.51	6.387	18.13	12.23	25.04	15.35	34.89
	±0.59	±1.31	±1.98	±0.71	±2.90	±0.05	±1.15	±0.28	±1.10	±2.05	±0.07	±0.14	±0.13	±0.27	±0.14	±0.82
CCB3	51.45	94.39	109.84	32.69	198.83	5.680	88.46	16.10	84.39	123.14	7.085	17.69	12.13	27.18	17.78	38.92
	±1.07	±1.98	±2.34	±0.88	±4.42	±0.10	±1.80	±0.36	±1.57	±2.58	±0.13	±0.55	±0.37	±0.59	±0.30	±1.16
CCB4	52.13	92.74	89.36	21.48	187.43	6.383	80.92	13.52	88.57	116.80	7.181	22.72	15.17	27.99	17.48	39.89
	±0.28	±0.41	±1.36	±0.83	±0.31	±0.03	±0.25	±0.19	±1.23	±0.16	±0.06	±0.18	±0.14	±0.17	±0.16	±1.18
CCB5	52.23	97.85	110.55	32.82	201.99	5.990	89.02	16.53	86.21	126.59	7.672	20.18	13.08	28.53	17.91	39.81

	±0.65	±1.00	±1.16	±0.11	±1.81	±0.10	±1.49	±0.11	±0.96	±1.19	±0.14	±0.57	±0.38	±0.40	±0.28	±1.13
CCB6	48.47	91.24	109.64	31.60	199.52	5.377	86.95	15.30	83.07	124.87	6.172	16.48	11.04	25.46	16.48	36.75
	±0.20	±0.44	±0.46	±0.22	±1.43	±0.05	±0.41	±0.07	±0.52	±0.77	±0.08	±0.42	±0.18	±0.17	±0.15	±0.57
CCB7	52.64	94.59	95.04	22.07	199.27	5.950	85.39	13.74	92.69	124.37	5.911	22.26	14.56	27.50	17.09	40.88
	±0.72	±1.17	±2.94	±1.52	±2.97	±0.06	±1.36	±0.49	±0.35	±1.94	±0.05	±0.26	±0.18	±0.34	±0.23	±0.43
CCB8	50.42	92.33	102.14	30.90	187.70	5.856	81.86	16.00	80.24	118.26	7.209	20.47	13.80	27.28	16.91	37.29
	±0.19	±0.60	±1.11	±0.58	±1.87	±0.05	±0.73	±0.18	±0.54	±1.04	±0.06	±0.11	±0.12	±0.24	±0.12	±3.23
CCC1	40.89	75.30	96.98	25.06	167.96	4.117	73.28	14.78	72.56	102.86	4.315	14.35	8.05	21.49	14.58	36.34
	±0.53	±1.27	±1.98	±0.54	±3.23	±0.06	±1.18	±0.28	±1.31	±2.14	±0.06	±0.07	±0.04	±0.28	±0.22	±0.38
CCC2	41.90	76.36	84.08	16.91	169.30	4.488	72.68	12.34	80.76	103.22	3.935	18.47	9.66	21.77	13.80	36.72
	±0.08	±0.26	±2.09	±0.92	±0.58	±0.06	±0.54	±0.36	±0.96	±0.65	±0.20	±0.07	±0.09	±0.09	±0.17	±0.37
CCC3	45.02	81.95	99.29	25.31	178.00	4.724	79.61	15.75	78.19	108.26	4.459	18.53	10.16	23.51	14.02	37.57
	±0.60	±1.15	±1.82	±0.54	±2.57	±0.06	±1.15	±0.30	±0.97	±1.68	±0.08	±0.24	±0.02	±0.34	±0.22	±0.33
CCC4	46.63	86.08	104.59	26.55	183.84	5.218	81.98	15.99	80.52	112.95	5.703	18.62	10.29	26.39	15.52	34.77
	±1.02	±2.01	±2.81	±0.68	±4.54	±0.12	±2.19	±0.39	±1.97	±2.95	±0.13	±0.56	±0.26	±0.53	±0.22	±1.65
CCC5	43.74	82.10	102.97	26.68	180.25	4.098	77.30	15.58	79.65	112.39	4.744	16.15	8.54	24.52	14.97	35.01
	±0.54	±1.10	±0.96	±0.36	±2.00	±0.06	±0.78	±0.25	±0.65	±1.13	±0.08	±0.37	±0.19	±0.21	±0.19	±0.29
CCC6	46.29	84.72	92.01	19.19	184.78	4.592	78.66	13.40	88.72	114.33	4.738	20.87	10.72	24.92	14.34	37.88
	±0.31	±0.51	±2.23	±0.94	±1.47	±0.02	±0.89	±0.39	±0.65	±1.17	±0.03	±0.15	±0.15	±0.23	±0.28	±0.69
CCC7	54.04	101.44	106.69	34.02	204.83	6.355	90.53	18.50	86.53	129.77	7.289	24.78	12.85	29.44	16.58	40.70
	±1.47	±3.02	±2.85	±1.42	±5.97	±0.12	±2.57	±0.53	±2.24	±3.88	±0.17	±0.45	±0.25	±0.79	±0.50	±0.96
CCC8	48.39	91.88	103.94	32.56	192.15	5.536	83.21	17.10	79.06	121.88	5.938	20.02	10.77	26.35	15.49	35.63
	±1.55	±3.03	±3.37	±1.18	±6.13	±0.16	±2.56	±0.61	±2.41	±3.94	±0.18	±0.58	±0.33	±0.87	±0.42	±0.45
CCC9	50.39	95.69	111.17	34.81	203.03	6.346	89.03	18.50	83.76	128.94	7.254	18.23	10.35	27.52	16.56	37.45
	±0.86	±2.07	±1.01	±1.09	±2.26	±0.08	±0.86	±0.19	±0.92	±1.01	±0.08	±0.37	±0.14	±0.32	±0.04	±1.09
CCPT1	34.73	64.40	68.63	19.96	146.05	5.281	64.76	11.57	70.87	83.37	3.895	13.24	9.34	17.05	10.86	25.56
	±0.29	±0.63	±1.37	±0.87	±1.20	±0.03	±0.58	±0.32	±0.78	±0.75	±0.06	±0.05	±0.12	±0.15	±0.18	±0.26
CCPT2	49.10	93.90	103.09	31.50	196.99	5.761	86.66	16.17	84.21	121.67	6.227	22.19	11.90	26.26	14.79	35.65
	±0.67	±1.65	±2.38	±1.09	±3.29	±0.06	±1.63	±0.42	±1.04	±2.19	±0.09	±0.22	±0.07	±0.45	±0.31	±0.48

CCPT3	52.80	99.90	107.35	34.64	201.89	6.139	90.73	19.63	84.59	126.46	7.134	22.83	12.09	28.68	16.31	38.59
	±0.25	±0.72	±0.34	±0.28	±0.85	±0.05	±0.23	±0.06	±0.44	±0.32	±0.07	±0.39	±0.19	±0.06	±0.30	±1.29
CCPT4	49.64	96.29	104.32	33.90	195.52	5.633	84.94	18.65	81.93	123.62	7.064	19.81	10.39	27.58	16.52	34.75
	±0.55	±0.66	±0.32	±0.42	±0.31	±0.08	±0.59	±0.24	±0.15	±0.31	±0.14	±0.66	±0.32	±0.27	±0.15	±1.87
CCPT5	46.37	88.92	95.21	37.18	189.85	7.278	87.87	19.16	87.39	106.30	7.539	17.80	13.05	24.34	13.93	33.25
	±3.20	±6.58	±6.75	±3.00	±13.20	±0.53	±6.07	±0.98	±6.12	±7.31	±0.28	±0.99	±0.90	±1.76	±1.11	±3.00
CCPT6	47.15	91.16	99.58	39.99	195.11	7.340	90.39	19.74	88.95	109.72	7.866	16.72	12.48	24.63	14.66	34.29
	±0.30	±0.47	±0.48	±0.11	±1.06	±0.07	±0.58	±0.13	±0.50	±0.51	±0.14	±0.37	±0.21	±0.22	±0.14	±1.30
CCPT7	45.57	88.94	99.44	40.35	193.81	7.101	88.40	19.23	87.81	109.43	7.298	14.62	11.08	24.04	15.00	32.78
	±0.85	±1.42	±1.26	±0.45	±2.60	±0.09	±1.44	±0.28	±1.24	±1.35	±0.11	±0.51	±0.35	±0.36	±0.15	±1.19
CCPT8	49.59	94.75	83.09	27.43	185.39	6.683	84.47	17.67	91.92	108.86	6.906	20.16	14.44	25.28	14.49	34.88
	±0.23	±0.66	±1.96	±1.08	±1.21	±0.12	±0.76	±0.64	±0.67	±0.97	±0.11	±0.07	±0.18	±0.29	±0.37	±0.30
CCPT9	49.51	93.91	88.09	28.61	198.54	6.471	89.22	17.53	95.38	116.16	6.428	19.69	13.40	23.24	13.93	34.26
	±1.29	±2.59	±1.38	±0.76	±5.25	±0.18	±2.32	±0.23	±2.63	±3.02	±0.19	±0.58	±0.27	±0.55	±0.32	±1.37
CCT1	49.22	95.35	99.79	40.68	203.10	5.874	91.84	20.78	90.86	120.06	6.301	18.12	13.21	23.73	13.85	34.72
	±2.21	±3.48	±2.36	±1.26	±4.70	±0.22	±2.42	±0.67	±2.29	±2.58	±0.15	±0.54	±0.34	±0.60	±0.40	±1.70
CCT2	50.70	96.60	104.49	43.21	207.54	6.116	94.80	22.65	91.29	122.47	5.752	16.59	12.42	23.47	14.86	36.74
	±0.95	±1.91	±2.51	±1.21	±4.31	±0.14	±2.10	±0.68	±1.85	±2.63	±0.19	±0.16	±0.12	±0.30	±0.39	±0.74
CCT3	51.37	98.54	106.81	45.13	211.49	6.346	94.76	22.45	92.63	127.66	6.208	15.12	11.11	24.16	15.44	36.03
	±0.93	±1.64	±1.71	±0.81	±3.39	±0.24	±1.83	±0.48	±1.44	±2.19	±0.17	±0.57	±0.42	±0.54	±0.33	±0.71
CCT4	54.63	105.17	99.71	32.15	229.83	8.056	105.05	18.48	109.53	135.44	5.680	21.82	14.98	26.26	14.94	41.07
	±0.15	±0.44	±2.57	±1.77	±1.19	±0.11	±0.76	±0.73	±0.81	±0.85	±0.19	±0.05	±0.24	±0.32	±0.21	±0.97
CCT5	52.31	100.36	102.17	42.50	210.06	7.023	99.29	22.26	95.84	122.24	6.656	20.22	14.73	24.57	14.14	41.35
	±0.57	±1.14	±1.93	±1.03	±2.92	±0.10	±1.73	±0.44	±1.07	±1.95	±0.12	±0.26	±0.20	±0.26	±0.29	±0.35
COC1	53.26	100.63	115.97	31.25	205.83	6.078	94.23	15.77	92.87	122.67	7.536	21.57	12.71	30.06	15.28	46.47
	±0.72	±1.64	±1.88	±0.65	±2.93	±0.11	±1.31	±0.43	±1.19	±1.86	±0.14	±0.31	±0.19	±0.57	±0.26	±0.12
COC2	51.96	98.85	116.24	31.54	205.27	5.736	91.99	15.59	92.18	122.66	7.295	18.59	10.77	29.50	15.45	44.53
	±0.71	±1.43	±1.76	±0.57	±2.85	±0.12	±1.47	±0.43	±1.23	±1.86	±0.26	±0.70	±0.44	±0.57	±0.38	±1.29
COC3	54.04	96.05	90.98	22.36	191.71	6.664	85.45	12.27	89.39	116.94	8.962	23.44	15.02	28.03	12.82	44.97

	±0.74	±1.38	±2.96	±1.33	±2.53	±0.12	±1.34	±0.68	±0.77	±1.60	±0.21	±0.32	±0.31	±0.60	±0.23	±1.63
CCS1	52.39	94.06	106.79	31.33	196.69	6.202	87.77	15.90	83.80	120.97	6.864	20.78	13.86	26.86	11.62	35.45
	±1.85	±3.45	±4.09	±1.37	±6.33	±0.21	±2.73	±0.70	±2.42	±4.29	±0.29	±0.43	±0.21	±0.66	±0.54	±8.51
CCS2	51.46	93.57	109.69	32.59	199.32	6.271	88.58	16.23	83.67	123.16	7.378	19.58	13.28	27.22	12.55	40.57
	±0.17	±0.28	±1.04	±0.04	±1.82	±0.07	±0.83	±0.31	±0.83	±1.13	±0.09	±0.46	±0.25	0.16	0.02	±0.84
CCS3	50.24	91.77	109.01	32.79	196.50	5.925	86.07	15.96	81.87	121.73	7.050	16.89	11.33	25.84	12.55	38.34
	±0.55	±0.62	±0.54	±0.10	±1.03	±0.03	±0.73	±0.08	±0.42	±0.51	±0.09	±0.43	±0.24	±0.06	±0.05	±0.85
CCS4	51.73	91.63	93.01	21.02	196.01	6.505	84.75	11.90	89.75	120.33	6.681	21.49	13.38	25.94	11.23	40.85
	±0.44	±1.16	±3.25	±1.48	±2.71	±0.18	±1.26	±0.45	±1.14	±2.09	±0.13	±0.22	±0.32	±0.55	±0.23	±1.06
EG1	60.35	112.16	136.63	34.93	243.55	6.582	104.81	22.30	102.60	146.97	4.688	23.18	14.62	30.11	12.85	53.72
	±0.48	±0.95	±1.54	±0.70	±1.81	±0.01	±0.46	±0.32	±0.51	±1.39	±0.11	±0.32	±0.15	±0.05	±0.10	±7.26
EG2	59.43	109.74	138.48	35.76	241.27	6.308	103.01	22.09	100.09	145.05	4.493	19.82	13.12	29.18	13.40	48.94
	±0.10	±0.14	±0.09	±0.11	±0.56	±0.08	±0.11	±0.33	±0.51	±0.44	±0.08	±0.40	±0.15	±0.07	±0.51	±0.40
EG3	57.62	107.15	137.95	35.34	238.84	5.724	100.18	22.08	98.42	143.69	3.974	16.78	10.91	29.00	13.79	46.49
	±0.50	±1.23	±1.36	±0.65	±2.71	±0.01	±0.65	±0.49	±1.31	±1.63	±0.08	±0.53	±0.23	±0.37	±0.31	±0.24
EG4	57.52	107.49	120.84	24.04	240.34	6.341	100.29	15.98	107.90	143.33	3.007	23.64	13.60	28.56	11.61	46.77
	±0.67	±1.45	±5.62	±2.41	±2.89	±0.07	±1.65	±0.65	±0.90	±1.69	±0.19	±0.27	±0.40	±0.60	±0.24	±1.09

Table S8. The content range of different types of amino acids in GCMs and EG (calculated after deducting dry weight of water content)

GCMs type	Content（mg/g）								
	Hyp	Gly	Ala	Pro	Total essential amino acids	Total non-essential amino acids	Total flavor amino acids	Total sweet amino acids	Total aromatic amino acids
CCA (*n*=23)	84.66 -111.3	168.9 -198.0	68.86 -89.62	104.0 -120.4	105.9 -145.4	568.3 -687.8	379.2 -456.8	392.2 -461.2	21.00 -26.11
CCB (*n*=8)	89.36 -110.6	182.2 -202.0	76.70 -92.69	113.5 -126.6	20.4 -136.8	624.1 -700.4	414.0 -463.8	422.2 -470.1	21.72 -25.58
CCC (*n*=9)	84.08 -111.2	168.0 -204.8	72.56 -88.72	102.9 -129.8	109.6 -142.8	566.4 -714.8	375.6 -470.7	387.0 -480.0	17.73 -23.87
CCPT (*n*=9)	68.63 -107.4	146.0 -201.9	70.87 -95.38	83.37 -126.5	87.62 -138.1	493.3 -704.3	330.8 -462.6	337.1 -473.4	14.75 -23.59
CCT (*n*=5)	99.71 -106.8	203.1 -229.8	90.86 -109.5	120.0 -135.4	124.32 -137.6	703.3 -785.5	458.7 -519.8	481.4 -533.5	20.15 -21.65
COC (*n*=3)	90.98 -116.2	191.7 -205.8	89.39 -92.87	116.9 -122.7	134.4 -141.	671.6 -714.4	453.0 -475.4	439.3 -474.5	21.78 -22.82
CCS (*n*=4)	93.01 -109.7	196.0 -199.3	81.87 -89.75	120.3 -123.2	120.9 -129.4	668.4 -686.0	440.0 -447.9	445.5 -461.2	17.91 -19.93
EG (*n*=4)	120.8 -138.5	238.8 -243.6	100.1 -102.6	143.3 -147.0	139.0.9 -156.8	790.3 -816.6	519.8 -536.2	537.9 -556.9	14.62 -17.89

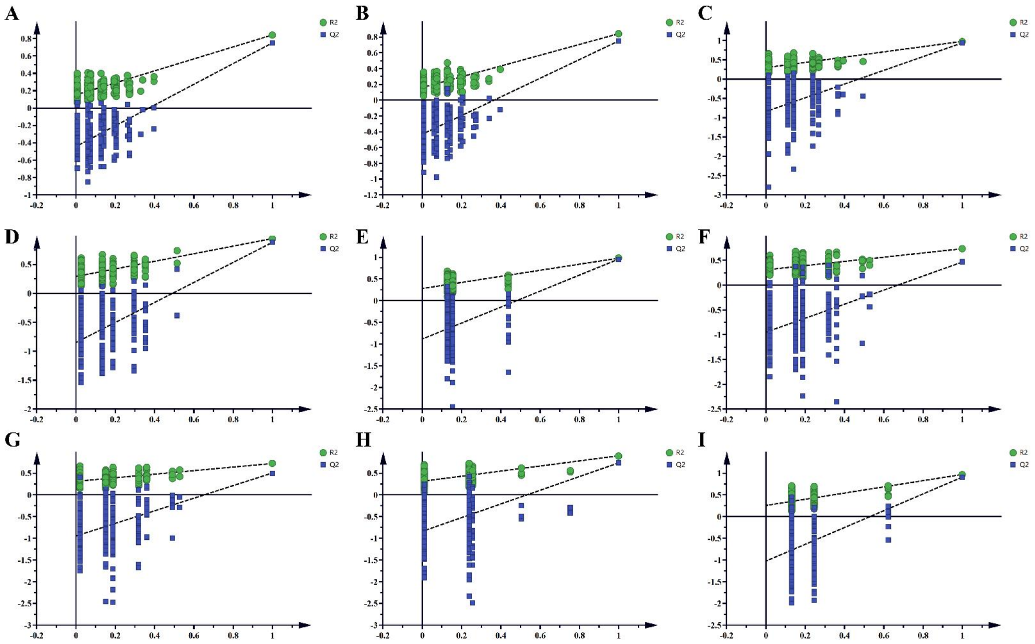

Figure S6. Permutation test results (n = 200) for the OPLS-DA models of different GCM groups: (A) dermal glues, (B) horn and shell glues, (C) CCA, (D) CCB, (E) CCS, (F) CCC, (G) CCPT, (H) CCT, and (I) COC.

Table S9. Targets of amino acid

Targets of amino acids					
EGLN1	GRIA2	BBOX1	ODC1	PTPRA	ADRA2A
GRIK1	GRM6	ABAT	KMO	NR1H4	ADRA2B
GRIA1	GRM2	GABBR2	NOS2	APEX1	ADRA1A
ADORA3	SLC1A2	GABBR1	NOS1	TAAR1	KDM1A
GRIK5	SLC6A12	ACE	ANPEP	KDM4C	CPA3
SLC1A1	SLC6A1	REN	F2	MAPKAPK2	SLC6A2
GRM4	GABRA1	CACNA2D1	LTA4H	KIF11	SLC6A4
GRM3	GABRB2	HRH3	KDM4E	ESR1	SLC15A1
GRIA4	GABRG2	HRH4	FYN	ESR2	PEPD
GRM5	GABRA3	CPB2	EGFR	CPB1	SHBG
GRM8	GABRA2	GFPT1	LCK	GRB2	AR
GRIK2	GABRR1	HRH2	TH	OAT	KYNU
GRIK3	SLC6A11	HRH1	SLC7A5	HDAC3	THRB
GRM1	SLC22A6	ENPEP	THRA	PLG	TACR1
GRM7	SLC6A13				

Table S10. Intersection target of "amino acid components-tonic activity"

"Amino acid components - tonic activity" intersection target					
GRIK1	GRM1	GABRG2	GABBR1	F2	TACR1
GRIA1	GRM7	GABRA3	ACE	FYN	ADRA2B
GRIK5	GRIA2	GABRA2	REN	EGFR	KDM1A
SLC1A1	SLC1A2	GABRR1	CACNA2D1	LCK	SLC6A4
GRM4	SLC6A12	SLC6A11	CPB2	TH	PEPD
GRIA4	SLC6A1	SLC6A13	GFPT1	ESR1	SHBG
GRM5	GABRA1	ABAT	NOS2	OAT	AR
GRIK2	GABRB2	GABBR2	NOS1	PLG	

Table S11. Topological properties of key components

Chemical compound	Degree	Betweenness	Closeness
Leu	22	0.0467	0.4724
Ile	21	0.0409	0.4651
Glu	20	0.0391	0.4511
Lys	17	0.0326	0.4255
Phe	13	0.0292	0.4138
Val	12	0.0131	0.4082

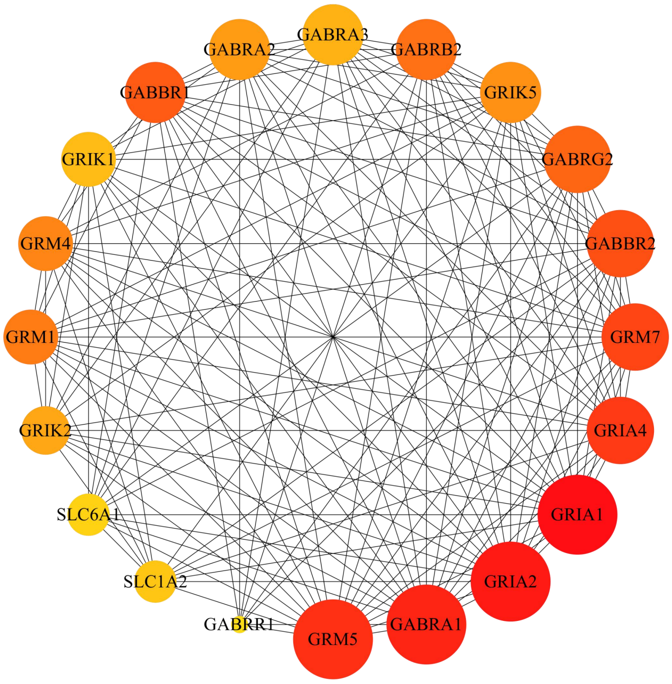

Figure S7. Protein-protein interaction diagram of intersection targets
